# Factors associated with deaths due to COVID-19 versus other causes: population-based cohort analysis of UK primary care data and linked national death registrations within the OpenSAFELY platform

**DOI:** 10.1016/j.lanepe.2021.100109

**Published:** 2021-07

**Authors:** Krishnan Bhaskaran, Sebastian Bacon, Stephen JW Evans, Chris J Bates, Christopher T Rentsch, Brian MacKenna, Laurie Tomlinson, Alex J Walker, Anna Schultze, Caroline E Morton, Daniel Grint, Amir Mehrkar, Rosalind M Eggo, Peter Inglesby, Ian J Douglas, Helen I McDonald, Jonathan Cockburn, Elizabeth J Williamson, David Evans, Helen J Curtis, William J Hulme, John Parry, Frank Hester, Sam Harper, David Spiegelhalter, Liam Smeeth, Ben Goldacre

**Affiliations:** a Faculty of Epidemiology and Population Health, London School of Hygiene and Tropical Medicine; b The DataLab, Nuffield Department of Primary Care Health Sciences, University of Oxford, Oxford, UK; c The Phoenix Partnership, TPP House, Horsforth, Leeds, UK; d Winton Centre for Risk and Evidence Communication, Statistical Laboratory Centre for Mathematical Sciences, Cambridge, UK

**Keywords:** COVID-19, Mortality, Epidemiology

## Abstract

**Background:**

Mortality from COVID-19 shows a strong relationship with age and pre-existing medical conditions, as does mortality from other causes. We aimed to investigate how specific factors are differentially associated with COVID-19 mortality as compared to mortality from causes other than COVID-19.

**Methods:**

Working on behalf of NHS England, we carried out a cohort study within the OpenSAFELY platform. Primary care data from England were linked to national death registrations. We included all adults (aged ≥18 years) in the database on 1^st^ February 2020 and with >1 year of continuous prior registration; the cut-off date for deaths was 9^th^ November 2020. Associations between individual-level characteristics and COVID-19 and non-COVID deaths, classified according to the presence of a COVID-19 code as the underlying cause of death on the death certificate, were estimated by fitting age- and sex-adjusted logistic models for these two outcomes.

**Findings:**

17,456,515 individuals were included. 17,063 died from COVID-19 and 134,316 from other causes. Most factors associated with COVID-19 death were similarly associated with non-COVID death, but the magnitudes of association differed. Older age was more strongly associated with COVID-19 death than non-COVID death (e.g. ORs 40.7 [95% CI 37.7-43.8] and 29.6 [28.9-30.3] respectively for ≥80 vs 50-59 years), as was male sex, deprivation, obesity, and some comorbidities. Smoking, history of cancer and chronic liver disease had stronger associations with non-COVID than COVID-19 death. All non-white ethnic groups had higher odds than white of COVID-19 death (OR for Black: 2.20 [1.96-2.47], South Asian: 2.33 [2.16-2.52]), but lower odds than white of non-COVID death (Black: 0.88 [0.83-0.94], South Asian: 0.78 [0.75-0.81]).

**Interpretation:**

Similar associations of most individual-level factors with COVID-19 and non-COVID death suggest that COVID-19 largely multiplies existing risks faced by patients, with some notable exceptions. Identifying the unique factors contributing to the excess COVID-19 mortality risk among non-white groups is a priority to inform efforts to reduce deaths from COVID-19.

**Funding:**

Wellcome, Royal Society, National Institute for Health Research, National Institute for Health Research Oxford Biomedical Research Centre, UK Medical Research Council, Health Data Research UK.

**Research in context**

**Evidence before this study**

A range of demographic and clinical risk factors for COVID-19 death were established in the first few months of the pandemic but little evidence is available on whether such factors have similar associations with COVID-19 death and non-COVID-19 death.

**Added value of this study**

We used data from a large database linking richly detailed primary care records and death registrations for 40% of the population of England. We conducted analyses comparing factors associated with COVID-19 and non-COVID deaths to generate unique insights into the extent to which risk factors for COVID-19 death mirror broader risk factors for death. We carried out a range of sensitivity analyses to ensure that our findings were robust.

**Implications of all the available evidence**

COVID-19 appears to largely act as if multiplying existing risks faced by patients. Public health decisions requiring prioritisation of vulnerable subgroups can therefore be informed by our knowledge of pre-pandemic mortality risks based on established risk factors. However there were some key exceptions; notably, higher risks of COVID-19 death for non-white ethnic groups were in contrast to lower risks of non-COVID deaths in these groups. Improved understanding of the unique drivers of COVID-19 mortality in non-white groups should be a research priority.

Alt-text: Unlabelled box

## 1 Introduction

Severe acute respiratory syndrome coronavirus 2 (SARS-CoV-2) has infected tens of millions of people worldwide, causing substantial mortality [1]. Numerous factors have emerged as being associated with a higher risk of severe outcomes and death from COVID-19 [2]. Mortality appears to rise exponentially with increasing age. Male sex, obesity, socioeconomic deprivation, and a number of comorbidities are also associated with higher risk. [3,4] Substantial variation in mortality by ethnicity has also been observed in several studies, with evidence from both the UK and US suggesting worse COVID-related outcomes among minority ethnic groups, compared with the majority White populations. [5], [6], [7] However, little evidence is available on how the factors associated with COVID-19 mortality compare with the factors associated with mortality from other causes, and hence the extent to which a person's risk of dying from COVID-19 is likely to be governed by their broader mortality risk. We know that increasing age is the major risk factor for all-cause mortality. It is possible that COVID-19 simply multiplies everyone's risk of death by a constant factor, or it could be that some factors have a different effect on COVID-19 deaths specifically. A better understanding of this would help inform strategies to identify and protect those most at risk of poor outcomes during the pandemic.

A previous analysis of death registration data in England and Wales showed exponential relationships between adult age and both rates of COVID-19 death (between March and June 2020), and pre-pandemic rates of all-cause mortality derived from life tables, with a slightly steeper age-mortality association for COVID-19 death [8]. A study using UK Biobank data found that both modifiable and non-modifiable risk factors for COVID-19 infection were somewhat stronger than for other infections, but did not assess severity of disease or mortality [9]. A further study using UK Biobank data from before the current pandemic examined how demographic characteristics and non-communicable disease comorbidities were associated with deaths from infections versus other causes; the authors observed broadly similar patterns of risk for the two outcomes, though the magnitude of associations differed [10]. However, it is unclear to what extent findings from pre-pandemic infection-related deaths can be used to draw conclusions about COVID-19.

To our knowledge, no study to date has directly compared factors associated with COVID-19 versus non-COVID deaths in the same cohort. We aimed to address this by conducting parallel analyses of COVID-19 and non-COVID death outcomes using population-based data from England within the OpenSAFELY platform.

## 2 Methods

### 2.1 Study design and study population

A retrospective cohort study was carried out within OpenSAFELY, a new data analytics platform in England created to address urgent COVID-19 related questions, which has been described previously [4]. We used routinely-collected electronic data from primary care practices using TPP SystmOne software, covering 2816 practices and approximately 40% of the population in England, linked to Office of National Statistics (ONS) death registrations. We included all adults (aged 18 years or over) alive and under follow-up on 1^st^ February 2020, and with at least one year of continuous GP registration prior to this date, to ensure that baseline data could be adequately captured. We excluded people with missing age, sex, or index of multiple deprivation, since these are likely to indicate poor data quality. For a secondary analysis of deaths prior to the pandemic, a second cohort was extracted comprising all adults alive and under follow-up on 1^st^ February 2019 and with at least one year of GP registration prior to that date (hereafter referred to as the “2019 cohort”). Finally, we compared directly those that died due to COVID-19 and those that died from other causes to assess associations between individual level factors and cause of death (analogous to a case-control analysis).

### 2.2 Outcome and covariates

The outcomes were COVID-19 death, and deaths from causes other than COVID-19 (hereafter “non-COVID death”). Cause of death was assigned using the underlying cause of death field (main/primary cause of death, coded in ICD-10) in the death registration. COVID-19 death was defined as any death with the underlying cause coded as U07.1 (“COVID-19, virus identified”) or U07.2 (“COVID-19, virus not identified”) [11]. Non-COVID deaths comprised all other deaths; these were also further sub-divided into categories covering the most common causes of death, namely cancer (ICD-10 chapter C), cardiovascular disease (chapter I), respiratory (chapter J), dementia/Alzheimer's disease (F00-03 or G30), and other (all other ICD-10 codes). Two sensitivity analyses were done to check that our findings were robust to the way COVID-19 deaths were defined: (i) only using the U07.1 (“virus identified”) code which would likely have higher specificity; (ii) counting a U07.1/U07.2 code anywhere on the death certificate as a COVID-19 death, in case of variation in how underlying causes were assigned. For the secondary analysis of deaths prior to the pandemic, the outcome was all-cause mortality; this was based on a record for death in primary care, because ONS death registration linkage for 2019 was not available.

Covariates considered in the analysis included health conditions listed in UK guidance on higher risk groups; [12] other common conditions that may cause immunodeficiency inherently or through medication, and other postulated risk factors for severe outcomes among COVID-19 cases. We included age (grouped as 18-39, 40-49, 50-59, 60-69, 70-79 and ≥80 years for descriptive analysis), sex, ethnicity (White, Mixed, South Asian, Black, Other, categories from the UK census), obesity (categorised as class I [body mass index 30-34.9kg/m^2^], II [35-39.9kg/m^2^], III [≥40kg/m^2^]), smoking status (never, former, current), index of multiple deprivation quintile (derived from the patient's postcode at lower super output area level). We also considered the following comorbidities: diagnosed hypertension, chronic respiratory diseases other than asthma, asthma (categorised as with or without recent use of oral steroids), chronic heart disease, diabetes (categorised according to the most recent glycated haemoglobin (HbA1c) recorded in the 15 months prior to 1^st^ February 2020), non-haematological and haematological cancer (both categorised by recency of diagnosis, <1, 1-4.9, ≥5 years), reduced kidney function (categorised by estimated glomerular filtration rate derived from the most recent serum creatinine measure (30-<60, 15-<30, <15 mL/min/1.73m^2^ or a record of dialysis), chronic liver disease, stroke, dementia, other neurological disease (motor neurone disease, myasthenia gravis, multiple sclerosis, Parkinson's disease, cerebral palsy, quadriplegia or hemiplegia, and progressive cerebellar disease), organ transplant, asplenia (splenectomy or a spleen dysfunction, including sickle cell disease), rheumatoid arthritis/lupus/psoriasis, and other immunosuppressive conditions (permanent immunodeficiency ever diagnosed, or aplastic anaemia or temporary immunodeficiency recorded within the last year). The Sustainability and Transformation Partnership (STP, an NHS administrative region) of the patient's general practice was included as an additional adjustment for geographical variation in infection rates across the country.

Information on all clinical covariates was obtained by searching TPP SystmOne records prior to 1^st^ February 2020 (or prior to 1^st^ February 2019, for the 2019 secondary analysis cohort) for specific coded data, based on a subset of SNOMED-CT mapped to Read version 3 codes. All codelists, along with detailed information on their compilation are available at https://codelists.opensafely.org for inspection and re-use by the wider research community.

### 2.3 Statistical analysis

Follow-up time for mortality in the main cohort was from 1^st^ February 2020 until 9^th^ November 2020, which was the last date for which mortality data were complete. Overall absolute probabilities of COVID-19 death and non-COVID death (by cause of death category) over this period were calculated for each age group, and standardised by sex; this was done by fitting a multinomial logistic regression model with outcome levels of died (by cause of death category) versus alive at end of follow-up, including covariates of age group and sex, and using fitted covariate and constant terms to predict risks for each outcome under a 50:50 male:female split [13]. Confidence intervals were computed by taking the 5^th^ and 95^th^ centiles of estimates from 1000 bootstrapped replications of this process.

To estimate differential associations between potential risk factors and mortality from COVID or non-COVID causes, binary logistic regression models were then fitted, with outcomes of (i) COVID-19 death and (ii) non-COVID death (all non-COVID causes combined). Logistic regression was chosen because the use of linked national mortality data meant that there was effectively a fixed follow-up period for outcome ascertainment; however a sensitivity analysis was also done using time-to-event Cox regression. For each outcome, separate models adjusted for age, sex, and STP were fitted for each covariate, with age parametrised as a 4-knot restricted cubic spline in regression models and STP included as a fixed effect. An additional model was fitted with age group sex, and STP only, to provide odds ratios for age group (since model estimates for spline terms are difficult to interpret). For the secondary analysis using the 2019 cohort, a similar set of age, sex and STP-adjusted logistic models was fitted using a follow-up time period from 1^st^ February to 9^th^ November 2019, with the outcome of all-cause mortality, which by definition represents non-COVID mortality during this pre-pandemic time period.

In further secondary analyses, models for each outcome were also fitted, adjusting for all covariates simultaneously to identify independent associations between covariates and outcomes. In a post-hoc analysis driven by the striking pattern of results for ethnicity, further age, sex, and STP-adjusted logistic models were fitted to explore ethnic differences in the odds of death from specific cause of death categories. Separate secondary analyses were done for wave 1 and 2 of the pandemic in England, based on the periods 1^st^ February-31^st^ August and 1^st^ September-9^th^ November respectively. Finally, to directly estimate the comparative association between individual-level factors and COVID-19 versus non-COVID deaths, we fitted age, sex, and STP-adjusted logistic models only including people who died, with cause of death (COVID-19 versus non-COVID) as the binary outcome variable. In these analyses, odds ratios >1 indicate that a variable has a more positive association with COVID-19 death than with non-COVID death, and vice versa.

Multiple imputation (10 imputations) was used to account for missing ethnicity, with the imputation model including all covariates from the main model and an indicator for the outcome. Those with missing body mass index were assumed to be non-obese, and those with missing smoking data were assumed to be never-smokers; we did not use multiple imputation for these variables as they are expected to be missing not at random in UK primary care [14]. In sensitivity analyses, we excluded individuals with missing data (complete case analysis).

### 2.4 Information governance and ethics

NHS England is the data controller; TPP is the data processor; and the key researchers on OpenSAFELY are acting on behalf of NHS England. OpenSAFELY is hosted within the TPP environment which is accredited to the ISO 27001 information security standard and is NHS IG Toolkit compliant; [15,16] patient data are pseudonymised for analysis and linkage using industry standard cryptographic hashing techniques; all pseudonymised datasets transmitted for linkage onto OpenSAFELY are encrypted; access to the platform is via a virtual private network (VPN) connection, restricted to a small group of researchers who hold contracts with NHS England and only access the platform to initiate database queries and statistical models. All database activity is logged. No patient-level data leave the platform; only aggregate statistical outputs leave the platform environment following best practice for anonymisation of results such as statistical disclosure control for low cell counts [17]. The OpenSAFELY platform adheres to the data protection principles of the UK Data Protection Act 2018 and the EU General Data Protection Regulation (GDPR) 2016. In March 2020, the Secretary of State for Health and Social Care used powers under the UK Health Service (Control of Patient Information) Regulations 2002 (COPI) to require organisations to process confidential patient information for the purposes of protecting public health, providing healthcare services to the public and monitoring and managing the COVID-19 outbreak and incidents of exposure [18]. Taken together, these provide the legal bases to link patient datasets on the OpenSAFELY platform. This study was approved by the Health Research Authority (REC reference 20/LO/0651) and by the LSHTM Ethics Board (ref 21863).

### 2.5 Role of the funding source

The funders had no role in study design, data collection, data analysis, interpretation, or writing of the report.

## 3 Results

**Fig. 1:**
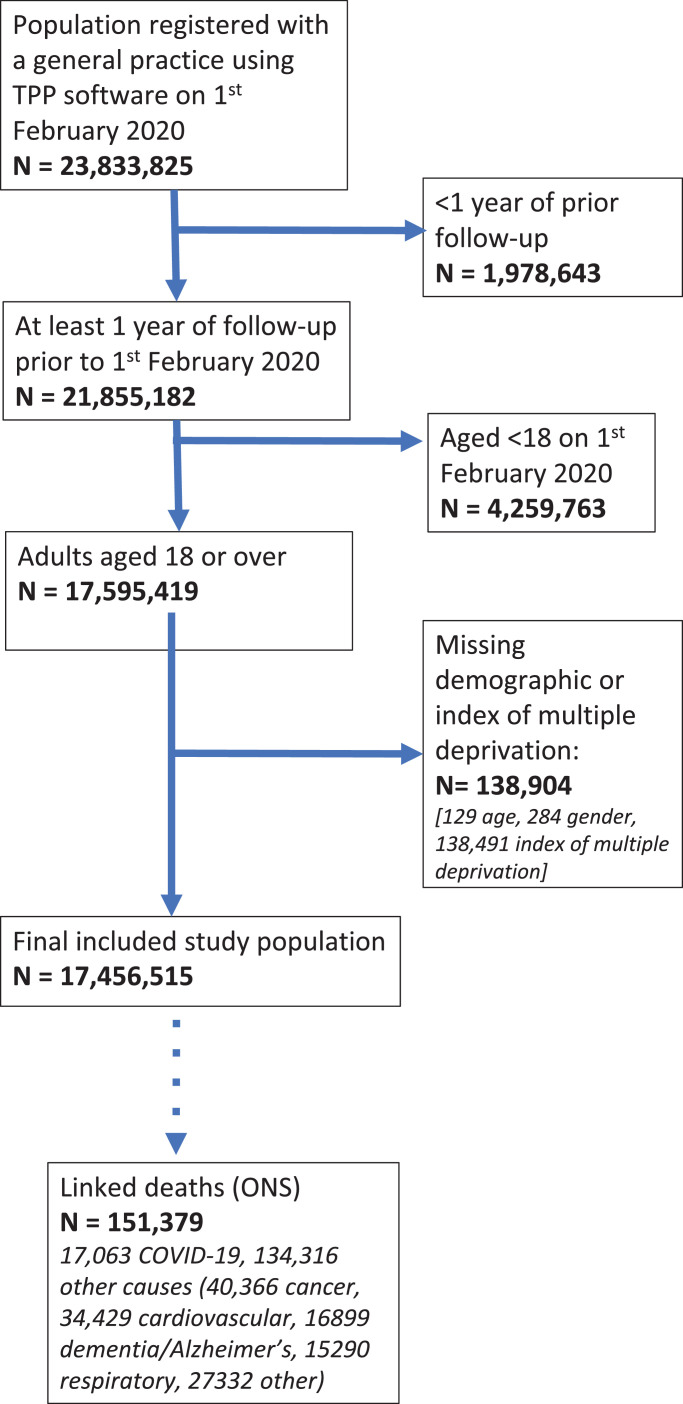
Flow chart of participants in the primary study cohort.

17,456,515 individuals were included in the analysis, of whom 17,063 died with COVID-19 listed as the underlying cause, while 134,316 died of other underlying causes (Figure 1). Demographic characteristics are shown in Table 1.

**Table 1:** Characteristics of the primary study cohort and distribution of COVID-19/non-COVID deaths

		N (%)	COVID-19 deaths	Non-COVID deaths
N		17456515 (100.0)	17063 (100.00)	134316 (100.00)
Age (yrs)	18-39	5965744 (34.2)	73 (0.43)	1346 (1.00)
	40-49	2877525 (16.5)	226 (1.32)	2711 (2.02)
	50-59	3085141 (17.7)	729 (4.27)	7487 (5.57)
	60-69	2421249 (13.9)	1631 (9.56)	15242 (11.35)
	70-79	1963205 (11.2)	3984 (23.35)	32093 (23.89)
	80+	1143651 (6.6)	10420 (61.07)	75437 (56.16)
Sex	Female	8739169 (50.1)	7617 (44.64)	67774 (50.46)
	Male	8717346 (49.9)	9446 (55.36)	66542 (49.54)
Body mass index (kg/m^2^)*	Not obese	13621506 (78.0)	12541 (73.50)	108236 (80.58)
	30-34.9 (Obese class I)	2419268 (13.9)	2761 (16.18)	16630 (12.38)
	35-39.9 (Obese class II)	940080 (5.4)	1152 (6.75)	6222 (4.63)
	≥40 (Obese class III)	475661 (2.7)	609 (3.57)	3228 (2.40)
Smoking status*	Never	8739756 (50.1)	5678 (33.28)	43947 (32.72)
	Former	5747053 (32.9)	10276 (60.22)	72206 (53.76)
	Current	2969706 (17.0)	1109 (6.50)	18163 (13.52)
Ethnicity	White	11163018 (63.9)	11280 (66.11)	90619 (67.47)
	Mixed	172141 (1.0)	77 (0.45)	353 (0.26)
	South Asian	1027068 (5.9)	951 (5.57)	2734 (2.04)
	Black	343094 (2.0)	308 (1.81)	1054 (0.78)
	Other	323893 (1.9)	145 (0.85)	549 (0.41)
	*Missing*	4427301 (25.4)	4302 (25.21)	39007 (29.04)
Index of Multiple Deprivation	1 (least deprived)	3519427 (20.2)	2882 (16.89)	25941 (19.31)
	2	3555666 (20.4)	3144 (18.43)	27357 (20.37)
	3	3515186 (20.1)	3258 (19.09)	27696 (20.62)
	4	3491534 (20.0)	3727 (21.84)	26996 (20.10)
	5 (most deprived)	3374702 (19.3)	4052 (23.75)	26326 (19.60)
Comorbidities				
Hypertension		5990510 (34.3)	12635 (74.05)	97064 (72.27)
Chronic respiratory disease		711370 (4.1)	3598 (21.09)	28461 (21.19)
Asthma	With no oral steroid use	2484264 (14.2)	1902 (11.15)	14054 (10.46)
	With oral steroid use	296251 (1.7)	545 (3.19)	3361 (2.50)
Chronic heart disease		1179367 (6.8)	6202 (36.35)	46465 (34.59)
Diabetes	With HbA1c<58 mmol/mol	1053215 (6.0)	3604 (21.12)	23929 (17.82)
	With HbA1c>=58 mmol/mol	491874 (2.8)	1937 (11.35)	10860 (8.09)
	With no recent HbA1c measure	196831 (1.1)	664 (3.89)	4636 (3.45)
Cancer (non-haematological)	Diagnosed < 1 year ago	81070 (0.5)	299 (1.75)	9754 (7.26)
	Diagnosed 1-4.9 years ago	237331 (1.4)	669 (3.92)	11671 (8.69)
	Diagnosed ≥5 years ago	547778 (3.1)	1788 (10.48)	17467 (13.00)
Haematological malignancy	Diagnosed < 1 year ago	8878 (0.1)	59 (0.35)	835 (0.62)
	Diagnosed 1-4.9 years ago	28130 (0.2)	168 (0.98)	1453 (1.08)
	Diagnosed ≥5 years ago	64022 (0.4)	272 (1.59)	2164 (1.61)
Reduced kidney function	Estimated GFR 30-60	1012185 (5.8)	6296 (36.90)	43973 (32.74)
	Estimated GFR 15-<30	60836 (0.3)	1014 (5.94)	7339 (5.46)
	Estimated GFR <15 or dialysis	31027 (0.2)	372 (2.18)	2516 (1.87)
Chronic liver disease		100844 (0.6)	266 (1.56)	3718 (2.77)
Dementia		41460 (0.2)	1334 (7.82)	6747 (5.02)
Stroke		367717 (2.1)	2937 (17.21)	19769 (14.72)
Other neurological disease		172055 (1.0)	1068 (6.26)	6761 (5.03)
Organ transplant		20194 (0.1)	94 (0.55)	494 (0.37)
Asplenia		28083 (0.2)	58 (0.34)	631 (0.47)
Rheumatoid arthritis/lupus/psoriasis		885573 (5.1)	1543 (9.04)	11263 (8.39)
Other immunosuppressive conditions		45307 (0.3)	70 (0.41)	650 (0.48)

⁎ missing BMI included in 'not obese' (n = 3,711,186 (21.3%); missing smoking included in 'never smoker' (n = 732,342 (4.2%)). “Data from 1^st^ February 2020 to 9^th^ November 2020”

**Fig. 2:**
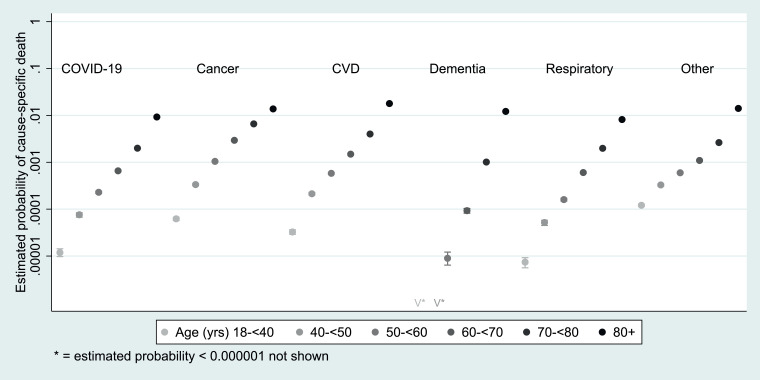
Estimated probability of death from different causes over the period between 1^st^ February and 9^th^ November 2020, by age group FOOTNOTES: Data from 1^st^ February 2020 to 9^th^ November 2020. Probabilities estimated from a multinomial logistic regression model with alive versus died from specific causes as outcomes, and with age group and sex fitted as covariates; estimates are standardised to a 50% male/female gender balance within each age group. Dementia includes Alzheimer's. CVD = cardiovascular diseases. For numerical estimates and 95% CIs please see appendix Table A1.

As expected, the probabilities of both COVID-19 death and death from other causes, among people in the general population during the 283-day study period were highly dependent on age. The probability of COVID-19 death over this period ranged from 0.0012% in those aged 18-39 years to 0.93% in those aged ≥80 years, similar to other respiratory causes combined (Figure 2, appendix Table A1). Probabilities of death from cardiovascular disease and cancer during this period were higher (ranging from 0.0033% to 1.8% in the youngest to oldest age groups for cardiovascular disease death, and from 0.0062% to 1.4% for cancer death); in those aged ≥80, the risk of dementia/ Alzheimer's death was 1.2%.

**Fig. 3:**
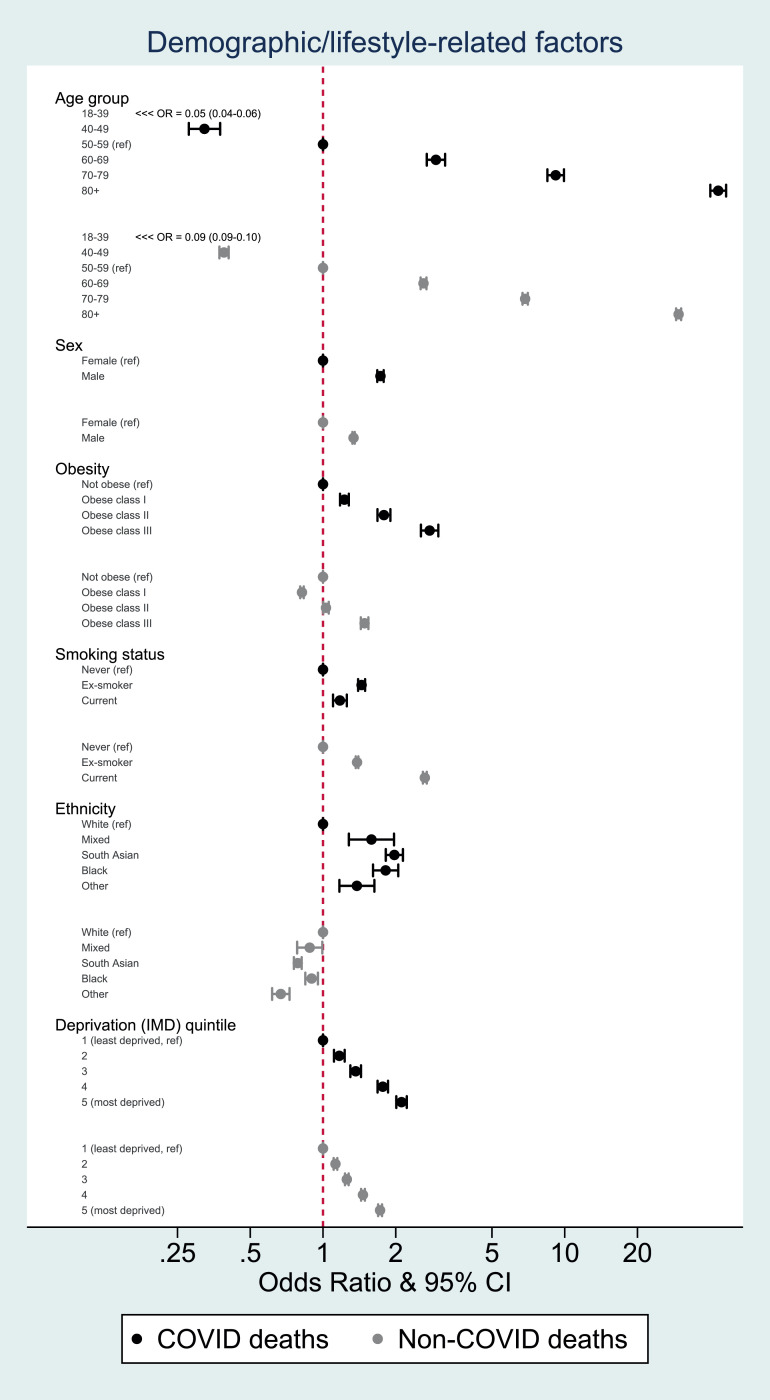
Odds ratios for the association between demographic and lifestyle-related factors and COVID-19 and non-COVID mortality, adjusted for age, sex and STP FOOTNOTES: Estimates for each covariate were produced by fitting two age, sex and STP-adjusted logistic models with outcomes of COVID-19 death and death from other causes respectively. Data from 1^st^ February 2020 to 9^th^ November 2020.

**Fig. 4:**
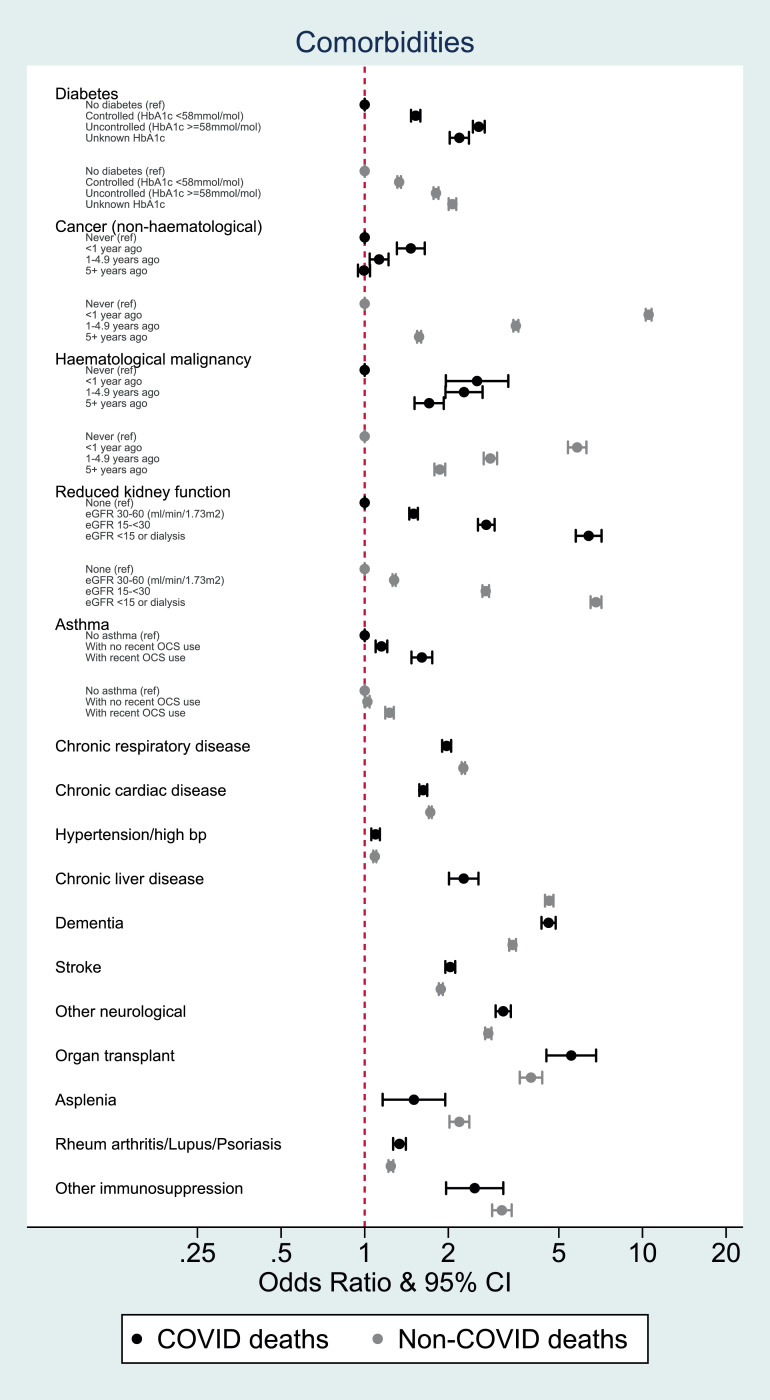
Odds ratios for the association between comorbidities and COVID-19 and non-COVID mortality, adjusted for age, sex and STP FOOTNOTES: Estimates for each covariate were produced by fitting two age, sex and STP-adjusted logistic models with outcomes of COVID-19 death and death from other causes respectively. Data from 1^st^ February 2020 to 9^th^ November 2020.

Most individual-level factors associated with odds of COVID-19 death had qualitatively similar associations with non-COVID death, but the magnitudes of association differed (Fig. 3, Fig. 4). Age, male gender, obesity, deprivation, and some comorbidities (notably uncontrolled diabetes, severe asthma, dementia and organ transplant) had more pronounced positive associations with COVID-19 death than non-COVID death. Smoking, history of cancer and chronic liver disease, while positively associated with both outcomes, had more pronounced positive associations with non-COVID death than COVID death.

**Fig. 5:**
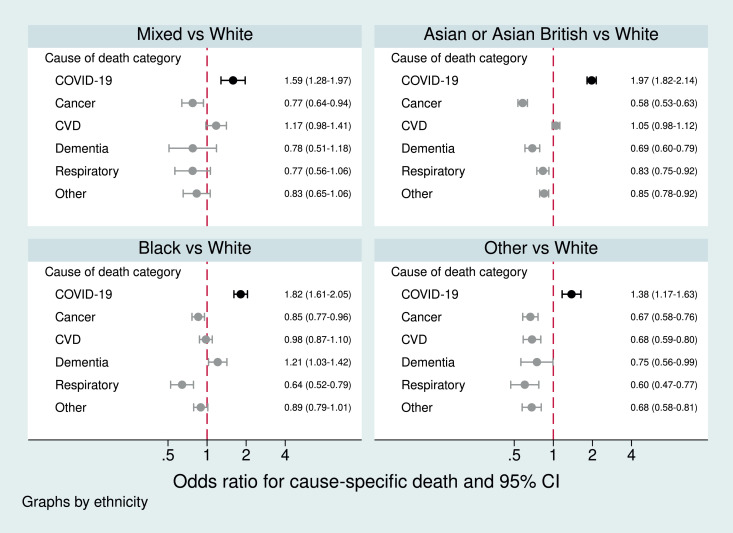
Odds ratios for the association between ethnicity and COVID-19 death and death from specific other causes, adjusted for age, sex, and STP FOOTNOTES:From separate logistic regression models for each cause-specific mortality outcome, with age (spline), sex, STP and ethnicity as covariates. Note: the dementia outcome included Alzheimer's and the model was restricted to those aged ≥40y due to non-convergence when younger people were included. Data from 1^st^ February 2020 to 9^th^ November 2020.

Non-white ethnicity had opposite associations with COVID-19 and non-COVID death: non-white ethnic groups had higher odds than white of COVID-19 death, but lower odds than white of death from non-COVID causes (Figure 3). This was also seen when non-COVID deaths were divided into more specific cause of death categories: non-white groups had similar or lower odds than white of death from cancer, cardiovascular disease, dementia/Alzheimer's, and respiratory causes of death (Figure 5).

**Fig. 6:**
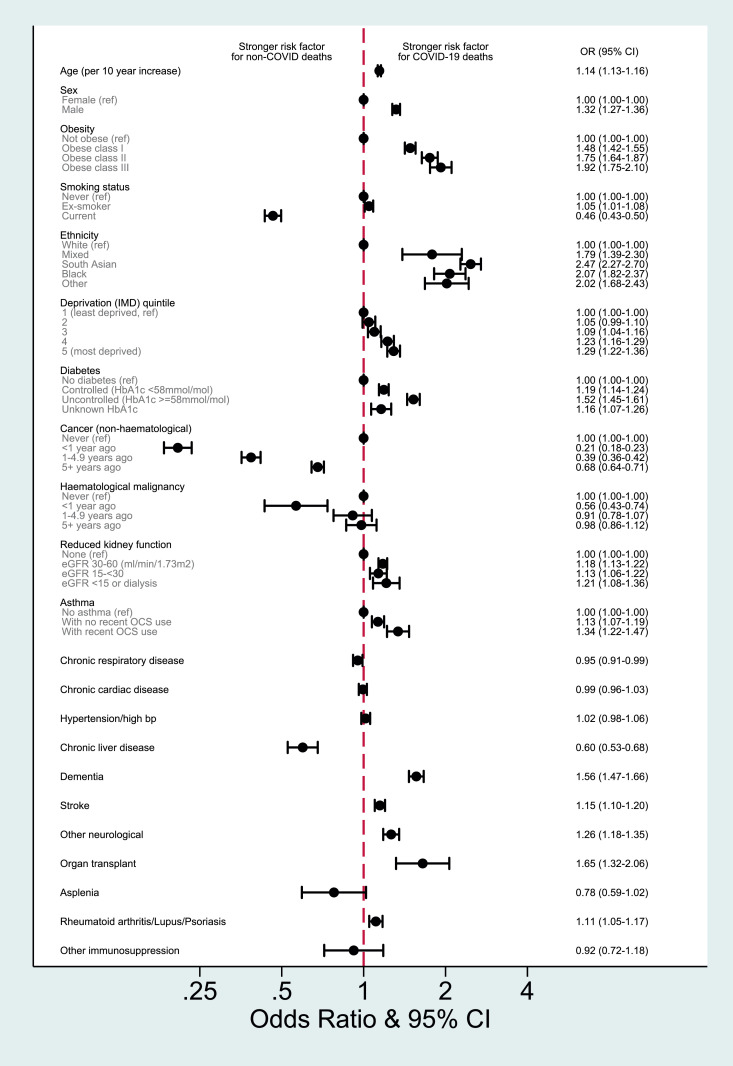
Odds ratio for COVID-19 cause of death (versus non-COVID causes) among those who died FOOTNOTES: Note that the odds ratio presented here are modelling the association between individual factors and the odds of a COVID-19 cause of death, among those who died. They cannot be interpreted as showing how factors are associated with the odds of death occurring (for this, see Fig. 3, Fig. 4). Estimates are from individual age, sex and STP-adjusted logistic regression models for each factor of interest, including only individuals that died, and with an outcome of COVID-19 cause of death. Age was parameterised as a 4-knot restricted cubic spline in all models, except to estimate the effect of age itself, where a linear age term was used for ease of presentation and interpretation. Data from 1^st^ February 2020 to 9^th^ November 2020.

An analysis restricted to those who died, directly modelling the odds of COVID-19 versus non-COVID cause of death, confirmed the pattern of results from our primary analyses (Figure 6). Older age, male sex, obesity, ethnicity, higher deprivation, diabetes, reduced kidney function, asthma, stroke, dementia, other neurological conditions, history of organ transplant, and autoimmune diseases were to varying degrees stronger risk factors for COVID-19 deaths, while smoking, haematological and non-haematological cancers, and chronic liver disease were stronger risk factors for non-COVID death.

Associations between individual-level factors and non-COVID deaths were similar when deaths from 2019 were used to represent non-COVID deaths, and patterns of results were also similar in analyses mutually adjusted for all variables (appendix Table A2). In analyses of separate pandemic waves, patterns of associations were mostly similar between the two waves, but the pattern of results for ethnicity changed in wave 2, with the South Asian group having continuing higher odds of COVID-19 death while other minority ethnic groups had similar odds to White; it was also notable that in contrast with wave 1, the second wave saw dementia having similar associations with COVID-19 and non-COVID death (appendix Figure A1(a-v). In sensitivity analyses, varying how COVID-19 deaths were defined, using complete case analysis to deal with missing data, and using a Cox regression modelling framework made little difference to the results (appendix Figure A1a-v).

## 4 Discussion

### 4.1 Key findings

Patterns of association between individual-level factors and risk of COVID-19 death largely mirrored those for non-COVID deaths, suggesting that COVID-19 has largely acted to multiplying existing mortality risks faced by patients. However, there were notable exceptions. People from non-white ethnic groups were at substantially raised risk of COVID-19 death compared with white people, despite having similar or lower risks of deaths from other causes. Several other demographic characteristics, lifestyle-related factors and comorbidities had qualitatively similar associations with risk of both COVID-19 and non-COVID death but with different magnitudes of association: age, male sex, obesity, deprivation and some comorbidities including severe asthma, uncontrolled diabetes, dementia and organ transplant, had stronger associations with COVID-19 deaths than non-COVID deaths. The opposite was true of smoking and other comorbidities including cancers and chronic liver disease, which were more strongly associated with non-COVID deaths. During the period from February to November 2020, COVID-19 was a common cause of death in England, though the incidence of cancer and cardiovascular disease deaths was higher in all age groups, and the incidence of deaths recorded as being due to dementia/Alzheimer's disease was also higher in the oldest individuals.

### 4.2 Findings in the context of other evidence

Although individual level factors were generally similarly associated with COVID-19 and non-COVID death, some of the observed differences were striking, including the discrepant effects of ethnicity on the two outcomes. A lower overall mortality risk in Black, South Asian and other minority ethnic groups has been observed before in an analysis of linked death registration data from 2001-2013 in Scotland, with suggested reasons including self-selection of healthy individuals among migrants, and healthier lifestyles and behaviours among these groups [19]. A study using pre-pandemic data from UK Biobank also observed a reduced risk of mortality in non-white ethnic groups that was consistent for both infectious and non-infectious deaths; [10] UK Biobank participants are not representative of the broader UK population, with evidence of a healthy volunteer selection bias, [20] and it is possible that this bias may have operated more strongly in non-white ethnic groups. Nevertheless the evidence from both the internal comparison in the present study, and related data from other studies, suggests that the higher risk of poor COVID-19 outcomes reflects unique features of the pandemic rather than a generalised higher risk of death in non-white groups. Reasons might include a high likelihood of working in at-risk occupations with high exposure risk, such as health and social care, hospitality and public transportation; and a high likelihood of living in large, high-density or multigenerational households, which might act individually or in combination to increase the risk of infection, and thus the overall risk of COVID-19 death, particularly if a high exposure risk in younger people leads to increased infection in older people via households and community settings [21]. Changes between wave 1 and 2 in the patterns of results for ethnicity are consistent with data recently published by ONS, [22] as well as a detailed analysis of OpenSAFELY focussing on associations between ethnicity and COVID-19 outcomes, [5] and suggest that ethnic differences in risk of COVID-19 death may be largely driven by exposure risk, which is more likely to have changed rapidly over time than susceptibility to severe disease.

Another notable finding was that, although current smoking was associated with a higher risk of both COVID-19 and non-COVID death, the association with COVID-19 death was substantially smaller than with non-COVID death. Current smokers also had a slightly smaller risk of COVID-19 death than ex-smokers, but the association was as expected for non-COVID death (current smokers at substantially higher risk than ex-smokers) suggesting that the finding was not driven by exposure misclassification or model mis-specification. Other studies have found smoking to be a significant risk factor for mortality among those hospitalised for COVID-19 [23]. If smokers have a raised risk of severe disease and death once infected, then it is possible that this has been diluted by a lower risk of infection in our general population-based study, but at present there is limited evidence to assess possible mechanisms that might explain such a reduced infection risk among smokers. Of note, the UK Biobank study found smoking to be more strongly associated with infection deaths than non-infection deaths in a pre-pandemic time period [10].

Among comorbidities, history of non-haematological cancer stood out as having a substantially smaller association with COVID-19 death compared with non-COVID death; cancer patients and survivors are likely to have a high long-term risk of cancer recurrence driving a raised risk of non-COVID (cancer) death; our results suggest that this is proportionately larger than the more modest raised risk of COVID-19 death that might arise from compromised immunity or risk of infection complications. Any underlying raised COVID-19 risk in this group may have been mitigated if cancer patients and survivors were more likely to shield and/or be compliant with social distancing measures. The difference between COVID-19 and non-COVID mortality risk was less stark in haematological cancer survivors, in keeping with evidence that these cancers are likely associated with a larger raised risk of COVID-19 death. [4,24] Chronic liver disease also showed a smaller association with COVID-19 death compared with non-COVID death, perhaps again reflecting shielding behaviour or social interactions during the pandemic; unfortunately we lacked the data to investigate the role of alcohol in this association. Dementia was associated with disproportionately raised risks of COVID-19 death, but this appeared to be driven by data from wave 1 of the pandemic, likely due to significant outbreaks in residential care homes during that period; by wave 2, dementia was associated with similar risks of COVID-19 and non-COVID death.

### 4.3 Strengths and limitations

Study strengths include the large size of the study, providing high statistical power to investigate associations between a wide range of factors and mortality. Our use of routinely collected primary care data meant that information on a wide range of longitudinal, detailed patient characteristics and comorbidities were available. Our data extraction and data management processes ensured that only records meeting data basic quality criteria were included; in addition, the underlying primary care data that we accessed are routinely extracted to support GP payment incentive schemes and national audits, ensuring that problems with data quality and flow are quickly addressed. Furthermore, individual-level linkage to death registrations provided near-complete ascertainment of mortality, with the caveat that a small proportion of deaths may have been missed among people who died outside the UK. UK primary care data have been shown to have good validity for ascertainment of a range of comorbidities, ethnicity and body mass index. [25], [26], [27] Our findings were robust in a number of sensitivity analyses.

There were also some limitations. Comorbidity ascertainment relied on conditions being coded in the primary care record; conditions will only be coded when patients consult, which may not happen for early-stage or mild illness. Conversely, acute conditions requiring hospitalisation may have been missed if feedback from hospital to primary care providers was imperfect. Missing data was an issue for some variables, notably ethnicity and body mass index. We used multiple imputation to deal with missing ethnicity, and our findings were robust to an alternative “complete case analysis” approach. Recording of body mass index in primary care is highly likely to be missing not at random, violating a key assumption required for multiple imputation, [14] so we instead assumed those with missing data to be non-obese, since obese individuals are more likely to have their weight recorded; however this could have caused some misclassification. Our results were again robust to an alternative “complete case approach”. We did not have any data on country of birth, to explore the effect of this variable and its interplay with ethnicity. We also lacked reliable information on some potentially important lifestyle-related variables such as alcohol use, diet and physical activity. We used the underlying cause of death field from the death registration to assign deaths as being due to COVID-19 or other causes, though results were similar in a sensitivity analysis where a COVID-19 death was defined based on a COVID-19 code anywhere on the death certificate. There may have been some misclassification of cause of death. Early in the epidemic, some COVID-19 deaths may have been misclassified as being due to other causes; for example a proportion of deaths due to COVID-19 occurring in care homes may have been misclassified as being due to dementia/Alzheimer's, biasing downwards our estimate of the absolute contribution of COVID-19 to overall mortality [28]. Conversely, during peak epidemic periods, a bias in the opposite direction may have occurred, if COVID-19 were entered as the presumed cause of death in uncertain cases. However, 16,049/17,063 COVID-19 deaths (94%) had the ICD-10 code U07.1 (“COVID-19 – virus identified”) implying that infection was confirmed by laboratory testing, so any misclassification in this direction is likely to have been minimal, and results were unchanged in a sensitivity analysis using an outcome definition restricted to this code. A caveat to this is that we could not be sure of the method of virus identification, as this was not part of the available data, or of the validity of the “COVID-19 – virus identified” code. We were further reassured given that we found patterns of results for non-COVID deaths in 2020 to be similar to those for pre-pandemic deaths in the equivalent period of 2019. COVID-19 mortality may have been affected by the competing risk of non-COVID death, and vice versa; our use of logistic regression over a fixed time period conceptually accounts for competing risks in a similar way to Fine & Gray modelling in a time to event framework, [29] since the estimated odds ratios target the association between covariate and odds of outcome, including any part of the association that is driven by the competing outcome. Our primary results describe associations between individual-level factors and outcomes adjusted for age, sex, and geography and in the supplementary appendix we also include results with mutual covariate adjustment to provide a more complete description of independent associations. However, we caution against interpreting our estimates as causal effects, which are challenging to estimate in observational data, and in this case would require the development of detailed variable-specific confounder models. In addition, since we estimated factors associated with COVID-19 death in the general population, rather than those with confirmed infection, it is not possible to disentangle from our data whether associations were driven by risk of infection, susceptibility to severe disease or a combination of the two. In the absence of widespread representative testing data, any attempt to look at factors associated with survival from infection would be highly vulnerable to collider bias because, for example, people with underlying ill health would be more likely to be tested and have infection ascertained, as well as being at increased risk of COVID-19 death [30]. Finally, TPP primary care software is not used uniformly around the country so our data are not fully geographically representative of England: in particular, the East of England, East Midlands, Yorkshire and the Humber regions are over-represented in the data, while London is underrepresented. Our adjustment for STP, which divided our data into 32 NHS administrative areas, should have avoided confounding by factors relating to geography in the estimation of associations, but observed absolute risks are not necessarily generalisable to England as whole.

### 4.4 Implications for public health and research

In all age groups, the probability of COVID-19 death was approximately similar to that for death from other respiratory causes combined during the time period of this study, but lower than the probability of death from cancer, cardiovascular disease and (among older individuals) dementia/Alzheimer's. This highlights the importance of maintaining care and prevention services targeting these high-burden non-COVID conditions. It should be remembered that the absolute probabilities of COVID-19 deaths we observed were specific to our study period, and were in the context of major national efforts to suppress infection rates and targeted shielding for particular risk groups. The relative contribution of COVID-19 to mortality would undoubtedly have been higher in the absence of these policies. The broadly similar relationships between most individual-level factors and COVID-19 and non-COVID mortality suggest that COVID-19 largely amplifies a person's underlying mortality risk (by a factor that will depend on current levels of circulating virus) based on their characteristics and medical history; public health decisions requiring prioritisation of vulnerable subgroups can therefore be informed by our knowledge of pre-pandemic mortality risks based on established risk factors. However there were important exceptions to this overall pattern. Understanding the drivers of uniquely raised risks of COVID-19 death associated with non-white ethnicity is a clear research priority; research into the role of occupations involving high levels of public contact, population density, and household composition will be key in further exploring the reasons, and thus informing mitigation strategies. Cancers were notably more strongly associated with non-COVID than COVID-19 deaths; a similar pattern was seen for chronic liver disease. However, this finding should not affect policies aimed at reducing risk in these groups, as even given a high underlying risk of non-COVID death, COVID-19 represents a potentially avoidable addition to the mortality risk. Emerging risk prediction tools can help to quantify this excess risk for individuals with different combinations of risk factors, and thus inform the targeting of mitigation measures, including prioritisation for vaccines. [3,31]

## 5 Conclusion

Demographic characteristics, lifestyle-related factors and comorbidities generally had qualitatively similar relationships with the risks of both COVID-19 death and non-COVID deaths, with some differences in the magnitudes of association. People from non-white ethnic groups had higher risks of COVID-19 death than white people, contrasting with similar or lower risks of deaths from other causes. This strongly suggests there are risk factors for mortality, specific to COVID-19, that are disproportionately affecting non-white groups; various factors along the causal chain culminating in COVID-19 death might contribute to the raised risks in non-white groups, including risk of exposure to the virus, risk of infection once exposed, underlying health and susceptibility to severe disease, health-seeking behaviour and health care received. Improved understanding of these factors is needed in order to tackle the increased mortality from COVID-19 among non-white groups. In conclusion, COVID-19 appears to largely act as if multiplying existing risks faced by patients, with the notable exceptions described in this paper.

## Contributors

BG conceived the OpenSAFELY platform and the approach. BG and LS lead the project overall and are guarantors. KB and SJWE led on study design. KB did the analysis and wrote the first draft. SB led on software development. AM led on information governance. Other contributions were – data curation - CB, JP, JC, SH, SB, DE, PI and CEM; disease category conceptualisation and codelists - KB CTR BM CB JC CEM AJW HIM IJD HJC JP; statistical analysis code: KB, EW; ethical approvals - HJC EW LS BG; software - SB, DE, PI, AJW, WJH, CEM, CB, FH, JC; writing (reviewing and editing) - KB, CTR, LT, BM, AS, AM, RME, CEM, IJD, SJWE, DS, LS, BG. All authors were involved in design and conceptual development and reviewed and approved the final manuscript.

## Declaration of interests

TPP provided technical expertise and infrastructure within their data centre *pro bono* in the context of a national emergency. BG's work on better use of data in healthcare more broadly is currently funded in part by: NIHR Oxford Biomedical Research Centre, NIHR Applied Research Collaboration Oxford and Thames Valley, the Mohn-Westlake Foundation, NHS England, and the Health Foundation; all DataLab staff are supported by BG's grants on this work. LS reports grants from Wellcome, MRC, NIHR, UKRI, British Council, GSK, British Heart Foundation, and Diabetes UK outside this work. KB held a Sir Henry Dale fellowship (grant: 107731/Z/15/Z) jointly funded by Wellcome and the Royal Society and a Wellcome Senior Research Fellowship (grant: 220283/Z/20/Z) during this work. HIM is funded by the National Institute for Health Research (NIHR) Health Protection Research Unit in Immunisation, a partnership between Public Health England and LSHTM. EW holds grants from MRC. ID golds grants from NIHR and GSK. HF holds a UKRI fellowship. RME is funded by HDR UK (grant: MR/S003975/1) and MRC (grant: MC_PC 19065). The views expressed are those of the authors and not necessarily those of the NIHR, NHS England, Public Health England or the Department of Health and Social Care. Funders had no role in the study design, collection, analysis, and interpretation of data; in the writing of the report; and in the decision to submit the article for publication.

## Data availability statement

All data were linked, stored and analysed securely within the OpenSAFELY platform https://opensafely.org/. Data include pseudonymized data such as coded diagnoses, medications and physiological parameters. No free text data are included. All code is shared openly for review and re-use under MIT open license (https://github.com/opensafely/covid-vs-noncovid-deaths-research). Detailed pseudonymised patient data are potentially re-identifiable and therefore not shared. We rapidly delivered the OpenSAFELY data analysis platform without prior funding to deliver timely analyses on urgent research questions in the context of the global Covid-19 health emergency: now that the platform is established we are developing a formal process for external users to request access in collaboration with NHS England; details of this process will be published on OpenSAFELY.org.

## Patient and Public Involvement

Patients were not formally involved in developing this specific study design that was developed rapidly in the context of a global health emergency. We have developed a publicly available website https://opensafely.org/ through which we invite any patient or member of the public to contact us regarding this study or the broader OpenSAFELY project.

## Appendix Supplementary materials

Image, application 1
