# Supplementary material for: Factors associated with deaths due to COVID-19 versus other causes: population-based cohort analysis of UK primary care data and linked national death registrations within the OpenSAFELY platform
Source: Lancet Reg Health Eur. 2021 May 8;6:100109. doi: 10.1016/j.lanepe.2021.100109 (PMC8106239; doi:10.1016/j.lanepe.2021.100109)
Supplement: Supplementary file 1 [file mmc1.pdf]

APPENDIX

Supplementary material for:

Bhaskaran K, Bacon SCJ, Evans SJW, Bates CJ, Rentsch CT, MacKenna B, Tomlinson L, Walker AJ, Schultze A, Morton CE, Grint D, Mehrkar A, Eggo RM, Inglesby P, Douglas IJ, McDonald HI, Cockburn J, Williamson EJ, Evans D, Curtis HJ, Hulme WJ, Parry J, Hester F, Harper S, Spiegelhalter D, Smeeth L, Goldacre B. Factors associated with deaths due to COVID-19 versus other causes: population-based cohort analysis of UK primary care data and linked national death registrations within the OpenSAFELY platform. *<Journal name> 2021*

Contents

Table A1: Estimated absolute risks of death from COVID-19 and non-COVID causes, stratified by age group

Table A2: Age, sex and STP adjusted and fully adjusted odds ratios for associations between individual-level factors and COVID-19/non-COVID deaths

Figure A1: Age, sex and STP adjusted odds ratios for associations between individual-level factors and COVID-19/non-COVID deaths in sensitivity analyses and analyses stratified by wave of pandemic

a – Age group

b – Sex

c – Obesity

d – Smoking

e – Ethnicity

f – Deprivation

g – Diabetes

h – Cancer (non-haematological)

i – Haematological malignancy

j – Reduced kidney function

k – Asthma

l – Chronic respiratory disease

m – Chronic cardiac disease

n – Hypertension/high blood pressure

o – Chronic liver disease

p – Dementia

q – Stroke

r – Other neurological disease

s – Organ transplant

t – Asplenia

u – Rheumatoid arthritis/lupus/psoriasis

v – Other immunosuppression

Table A1: Estimated absolute risks of death from COVID-19 and non-COVID causes, stratified by age group

	**Risk of death (%) over the period 1st February to 9th November 2020 (rounded to two significant figures)**					
Age group (years)	COVID-19	Cancer	Cardiovascular disease	Dementia/ Alzheimer's	Respiratory disease	Other
18-39	$1.2x10^{-3}$ ($9.7x10^{-4}$-$1.4x10^{-3}$)	$6.2x10^{-3}$ ($5.7x10^{-3}$-$6.7x10^{-3}$)	$3.3x10^{-3}$ ($2.9x10^{-3}$-$3.6x10^{-3}$)	$3.0x10^{-11}$ ($2.4x10^{-11}$-$6.5x10^{-11}$)	$7.4x10^{-4}$ ($5.6x10^{-4}$-$9.2x10^{-4}$)	0.012 (0.011-0.013)
40-49	$7.6x10^{-3}$ ($6.7x10^{-3}$-$8.4x10^{-3}$)	0.033 (0.032-0.035)	0.021 (0.02-0.023)	$6.9x10^{-5}$ ($3.0x10^{-11}$-$1.7x10^{-4}$)	$5.2x10^{-3}$ ($4.5x10^{-3}$-$5.8x10^{-3}$)	0.033 (0.031-0.034)
50-59	0.023 (0.022-0.024)	0.10 (0.10-0.11)	0.058 (0.056-0.060)	$9.0x10^{-4}$ ($6.4x10^{-4}$-$1.2x10^{-3}$)	0.016 (0.015-0.017)	0.06 (0.058-0.062)
60-69	0.066 (0.063-0.068)	0.29 (0.29-0.30)	0.15 (0.14-0.15)	$9.3x10^{-3}$ ($8.2x10^{-3}$-0.010)	0.060 (0.058-0.063)	0.11 (0.11-0.11)
70-79	0.20 (0.19-0.21)	0.66 (0.65-0.67)	0.4 (0.39-0.41)	0.10 (0.098-0.10)	0.20 (0.19-0.20)	0.26 (0.26-0.27)
≥80	0.93 (0.91-0.94)	1.4 (1.4-1.4)	1.8 (1.8-1.8)	1.2 (1.2-1.2)	0.82 (0.80-.83)	1.4 (1.4-1.4)

Table A2: Age-sex adjusted and fully adjusted odds ratios for associations between individual-level factors and COVID-19/non-COVID deaths

	Age-sex adjusted OR (95% CI)			Fully adjusted OR* (95% CI)		
	COVID-19 deaths 2020	Non-COVID deaths 2020	Deaths 2019	COVID-19 deaths 2020	Non-COVID deaths 2020	Deaths 2019
**Age**						
18-<40	0.05 (0.04-0.06)	0.09 (0.09-0.10)	0.15 (0.14-0.15)	0.06 (0.04-0.07)	0.11 (0.11-0.12)	0.18 (0.17-0.19)
40-<50	0.32 (0.28-0.38)	0.39 (0.37-0.41)	0.44 (0.42-0.46)	0.34 (0.29-0.39)	0.44 (0.42-0.46)	0.50 (0.48-0.52)
50-<60	1.00 (ref)	1.00 (ref)	1.00 (ref)	1.00 (ref)	1.00 (ref)	1.00 (ref)
60-<70	2.93 (2.69-3.20)	2.60 (2.53-2.68)	2.49 (2.43-2.56)	2.57 (2.35-2.80)	2.14 (2.09-2.21)	2.03 (1.98-2.09)
70-<80	9.17 (8.48-9.93)	6.86 (6.69-7.03)	6.44 (6.28-6.60)	6.74 (6.21-7.31)	4.48 (4.37-4.60)	4.12 (4.01-4.23)
80+	43.16 (40.03-46.53)	29.60 (28.90-30.31)	27.16 (26.52-27.81)	24.10 (22.23-26.13)	15.51 (15.11-15.93)	14.09 (13.72-14.46)
**Sex**						
Female	1.00 (ref)	1.00 (ref)	1.00 (ref)	1.00 (ref)	1.00 (ref)	1.00 (ref)
Male	1.73 (1.68-1.78)	1.34 (1.32-1.35)	1.38 (1.36-1.39)	1.55 (1.50-1.60)	1.11 (1.09-1.12)	1.13 (1.12-1.14)
**BMI**						
Not obese	1.00 (ref)	1.00 (ref)	1.00 (ref)	1.00 (ref)	1.00 (ref)	1.00 (ref)
30-34.9kg/m2 (Obese class I)	1.23 (1.18-1.28)	0.82 (0.80-0.83)	0.83 (0.82-0.85)	1.07 (1.03-1.12)	0.74 (0.73-0.75)	0.76 (0.75-0.77)
35-39.9kg/m2 (Obese class II)	1.79 (1.68-1.90)	1.03 (1.00-1.06)	1.04 (1.01-1.07)	1.44 (1.36-1.54)	0.87 (0.85-0.90)	0.89 (0.86-0.91)
≥40 kg/m2 (Obese class III)	2.76 (2.54-3.00)	1.49 (1.43-1.54)	1.46 (1.40-1.51)	2.11 (1.93-2.29)	1.21 (1.17-1.26)	1.20 (1.16-1.25)
**Smoking**						
Never	1.00 (ref)	1.00 (ref)	1.00 (ref)	1.00 (ref)	1.00 (ref)	1.00 (ref)
Former	1.44 (1.40-1.49)	1.38 (1.37-1.40)	1.41 (1.39-1.43)	1.26 (1.22-1.30)	1.14 (1.13-1.16)	1.15 (1.13-1.16)
Current	1.17 (1.10-1.25)	2.64 (2.59-2.69)	2.73 (2.68-2.78)	0.97 (0.91-1.04)	2.01 (1.97-2.05)	2.06 (2.02-2.11)
**Ethnicity**						
White	1.00 (ref)	1.00 (ref)	1.00 (ref)	1.00 (ref)	1.00 (ref)	1.00 (ref)
Mixed	1.59 (1.28-1.97)	0.88 (0.78-0.99)	0.88 (0.78-1.00)	1.43 (1.15-1.78)	0.85 (0.76-0.96)	0.86 (0.76-0.97)
South Asian	1.97 (1.82-2.14)	0.79 (0.76-0.82)	0.77 (0.74-0.80)	1.70 (1.55-1.85)	0.76 (0.73-0.79)	0.76 (0.73-0.79)
Black	1.82 (1.61-2.05)	0.90 (0.85-0.95)	0.79 (0.74-0.85)	1.44 (1.27-1.63)	0.81 (0.77-0.87)	0.72 (0.67-0.78)
Other	1.38 (1.17-1.63)	0.67 (0.62-0.73)	0.63 (0.58-0.69)	1.38 (1.16-1.63)	0.68 (0.62-0.74)	0.66 (0.60-0.72)
**IMD quintile**						
1 (least deprived)	1.00 (ref)	1.00 (ref)	1.00 (ref)	1.00 (ref)	1.00 (ref)	1.00 (ref)
2	1.17 (1.11-1.23)	1.13 (1.11-1.15)	1.12 (1.10-1.14)	1.13 (1.07-1.19)	1.09 (1.07-1.11)	1.09 (1.07-1.11)
3	1.37 (1.30-1.44)	1.25 (1.23-1.28)	1.25 (1.23-1.28)	1.25 (1.19-1.32)	1.18 (1.16-1.21)	1.18 (1.16-1.20)
4	1.77 (1.68-1.86)	1.46 (1.44-1.49)	1.44 (1.41-1.47)	1.53 (1.46-1.61)	1.33 (1.31-1.36)	1.30 (1.28-1.33)
5 (most deprived)	2.11 (2.01-2.22)	1.72 (1.69-1.75)	1.70 (1.67-1.74)	1.71 (1.62-1.80)	1.47 (1.44-1.50)	1.44 (1.41-1.46)
**Blood pressure**						
Normal	1.00 (ref)	1.00 (ref)	1.00 (ref)	1.00 (ref)	1.00 (ref)	1.00 (ref)
High bp or diagnosed hyper-tension	1.09 (1.06-1.13)	1.09 (1.07-1.10)	1.08 (1.06-1.09)	0.90 (0.87-0.94)	0.99 (0.97-1.00)	0.97 (0.96-0.99)
Co-morbidities						
**Respiratory disease ex asthma**	1.97 (1.90-2.05)	2.27 (2.23-2.30)	2.46 (2.43-2.50)	1.66 (1.59-1.73)	1.81 (1.78-1.83)	1.94 (1.91-1.97)
**Asthma (vs none)**						
With no recent OCS use	1.15 (1.10-1.21)	1.02 (1.00-1.04)	1.04 (1.03-1.06)	1.00 (0.95-1.05)	0.93 (0.92-0.95)	0.95 (0.94-0.97)
With recent OCS use	1.61 (1.47-1.75)	1.23 (1.19-1.27)	1.30 (1.25-1.35)	1.15 (1.05-1.26)	0.90 (0.87-0.94)	0.94 (0.90-0.97)
**Chronic heart disease**	1.62 (1.57-1.68)	1.72 (1.70-1.74)	1.80 (1.78-1.83)	1.23 (1.19-1.27)	1.42 (1.40-1.44)	1.48 (1.46-1.49)
**Diabetes (vs none)**						

With HbA1c<58 mmol/mol	1.53 (1.47-1.59)	1.33 (1.31-1.35)	1.37 (1.35-1.39)	1.20 (1.16-1.25)	1.16 (1.14-1.17)	1.18 (1.16-1.20)
WithHbA1c>=58 mmol/mol	2.57 (2.45-2.70)	1.81 (1.77-1.84)	1.84 (1.81-1.89)	1.83 (1.74-1.93)	1.53 (1.50-1.57)	1.55 (1.52-1.59)
With no recent HbA1c measure	2.19 (2.02-2.37)	2.07 (2.01-2.14)	2.05 (1.98-2.12)	1.71 (1.58-1.86)	1.82 (1.76-1.88)	1.77 (1.71-1.83)
**Cancer (non-haematological, vs none)**						
Diagnosed < 1 year ago	1.47 (1.31-1.65)	10.52 (10.27-10.78)	10.34 (10.09-10.59)	1.44 (1.28-1.62)	10.44 (10.18-10.70)	10.33 (10.08-10.59)
Diagnosed 1-4.9 years ago	1.13 (1.04-1.22)	3.50 (3.43-3.57)	3.53 (3.45-3.60)	1.11 (1.03-1.20)	3.48 (3.40-3.55)	3.49 (3.42-3.57)
Diagnosed ≥5 years ago	0.99 (0.95-1.04)	1.57 (1.54-1.60)	1.61 (1.58-1.64)	0.98 (0.94-1.03)	1.54 (1.52-1.57)	1.58 (1.55-1.60)
**Haematological malignancy (vs none)**						
Diagnosed < 1 year ago	2.54 (1.96-3.29)	5.82 (5.39-6.28)	5.39 (4.98-5.83)	2.41 (1.86-3.13)	5.00 (4.61-5.42)	4.58 (4.22-4.97)
Diagnosed 1-4.9 years ago	2.28 (1.95-2.66)	2.83 (2.68-3.00)	2.76 (2.60-2.92)	2.25 (1.92-2.62)	2.60 (2.45-2.75)	2.49 (2.35-2.65)
Diagnosed ≥5 years ago	1.71 (1.51-1.93)	1.86 (1.78-1.95)	2.01 (1.92-2.10)	1.65 (1.46-1.87)	1.71 (1.63-1.79)	1.86 (1.77-1.95)
**Reduced kidney function**						
Estimated GFR 30-60	1.50 (1.45-1.55)	1.28 (1.26-1.29)	1.27 (1.26-1.29)	1.30 (1.25-1.35)	1.15 (1.14-1.17)	1.14 (1.13-1.16)
Estimated GFR 15-<30	2.74 (2.56-2.93)	2.73 (2.65-2.80)	2.73 (2.66-2.81)	2.03 (1.90-2.18)	2.22 (2.16-2.28)	2.20 (2.14-2.26)
Estimated GFR <15 or dialysis	6.40 (5.75-7.12)	6.80 (6.50-7.12)	7.26 (6.95-7.60)	4.42 (3.93-4.98)	5.94 (5.65-6.24)	6.17 (5.86-6.49)
**Liver disease**	2.27 (2.01-2.57)	4.62 (4.46-4.78)	4.85 (4.68-5.03)	1.75 (1.54-1.98)	3.61 (3.48-3.74)	3.73 (3.59-3.87)
**Dementia**	4.59 (4.33-4.87)	3.41 (3.31-3.50)	3.20 (3.11-3.30)	3.62 (3.41-3.84)	2.98 (2.89-3.06)	2.79 (2.71-2.88)
**Stroke**	2.03 (1.95-2.12)	1.88 (1.85-1.91)	1.89 (1.86-1.92)	1.53 (1.46-1.59)	1.48 (1.46-1.51)	1.48 (1.45-1.50)
**Other neurological disease**	3.15 (2.96-3.36)	2.79 (2.71-2.86)	2.79 (2.71-2.86)	2.72 (2.55-2.90)	2.63 (2.56-2.70)	2.63 (2.56-2.71)
**Organ transplant**	5.54 (4.51-6.81)	3.97 (3.62-4.36)	4.45 (4.06-4.87)	1.61 (1.28-2.02)	0.83 (0.75-0.92)	0.90 (0.82-1.00)
**Asplenia**	1.50 (1.16-1.95)	2.19 (2.02-2.38)	2.15 (1.98-2.34)	1.26 (0.97-1.64)	1.62 (1.48-1.76)	1.56 (1.43-1.70)
**Rheumatoid/Lupus/ Psoriasis**	1.33 (1.27-1.41)	1.24 (1.22-1.27)	1.27 (1.25-1.30)	1.23 (1.17-1.30)	1.11 (1.09-1.14)	1.13 (1.11-1.16)
**Other immunosuppressive condition**	2.49 (1.96-3.15)	3.12 (2.88-3.38)	3.23 (2.98-3.51)	2.00 (1.57-2.54)	2.30 (2.11-2.50)	2.32 (2.13-2.53)

*Fully adjusted ORs are mutually adjusted for all factors in the table. All models additionally adjusted for STP.

Figure A1: Age-sex adjusted odds ratios for associations between individual-level factors and COVID-19/non-COVID deaths in sensitivity analyses and analyses stratified by wave of pandemic

(a)

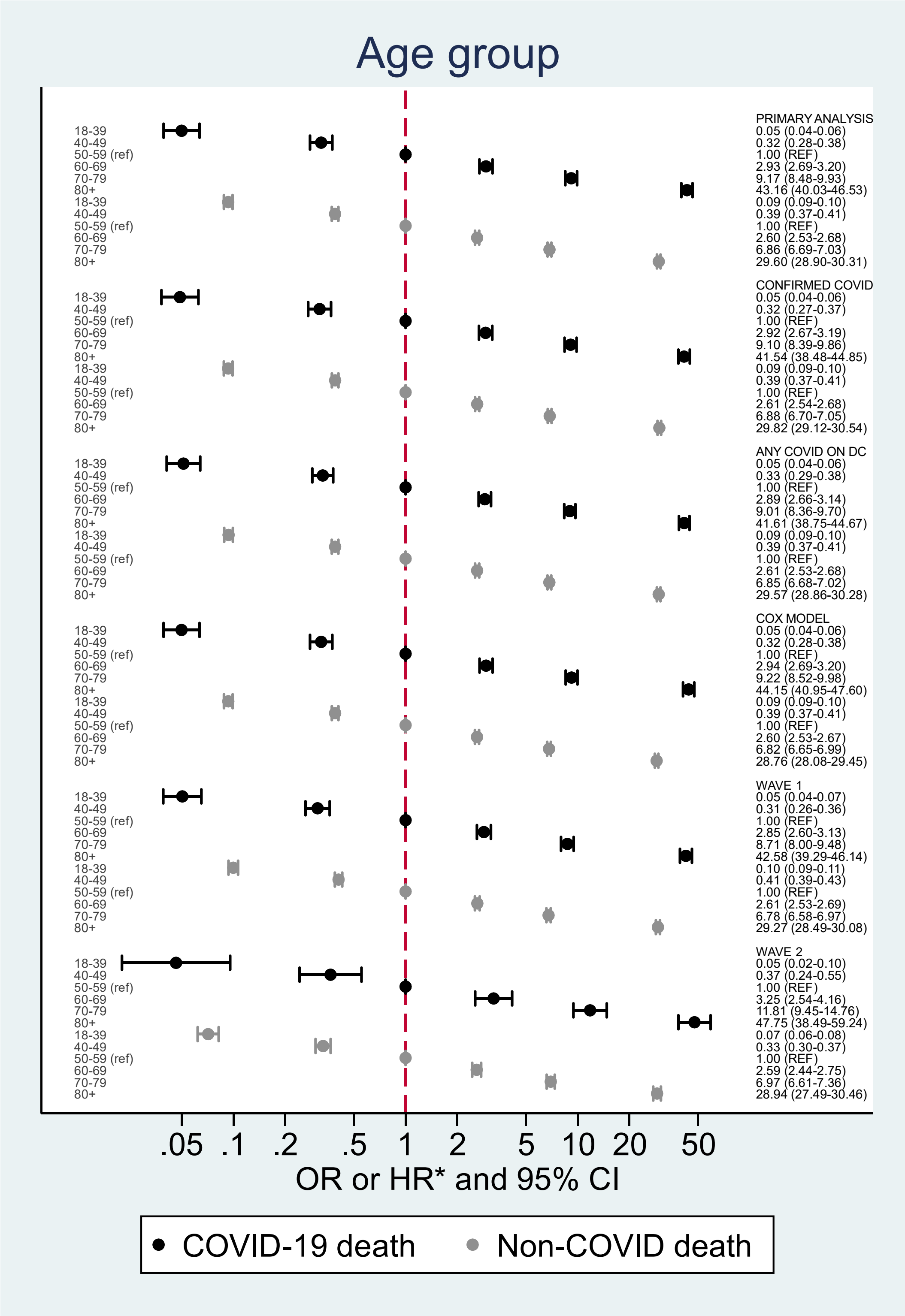

*CONFIRMED COVID = COVID-19 outcome defined using only the ICD-10 code U07.1 (COVID-19 – virus identified); ANY COVID ON DC = COVID-19 outcome defined as a COVID-19 code anywhere on the death certificate, rather than only as the underlying cause of death; COX MODEL = Hazard ratios from a Cox regression model (containing age / sex / covariate of interest and stratified by STP); WAVE 1 = 1st February to 31st August; WAVE 2 = 1st September to 9th November. *HR for the Cox model results, OR for all other results.*

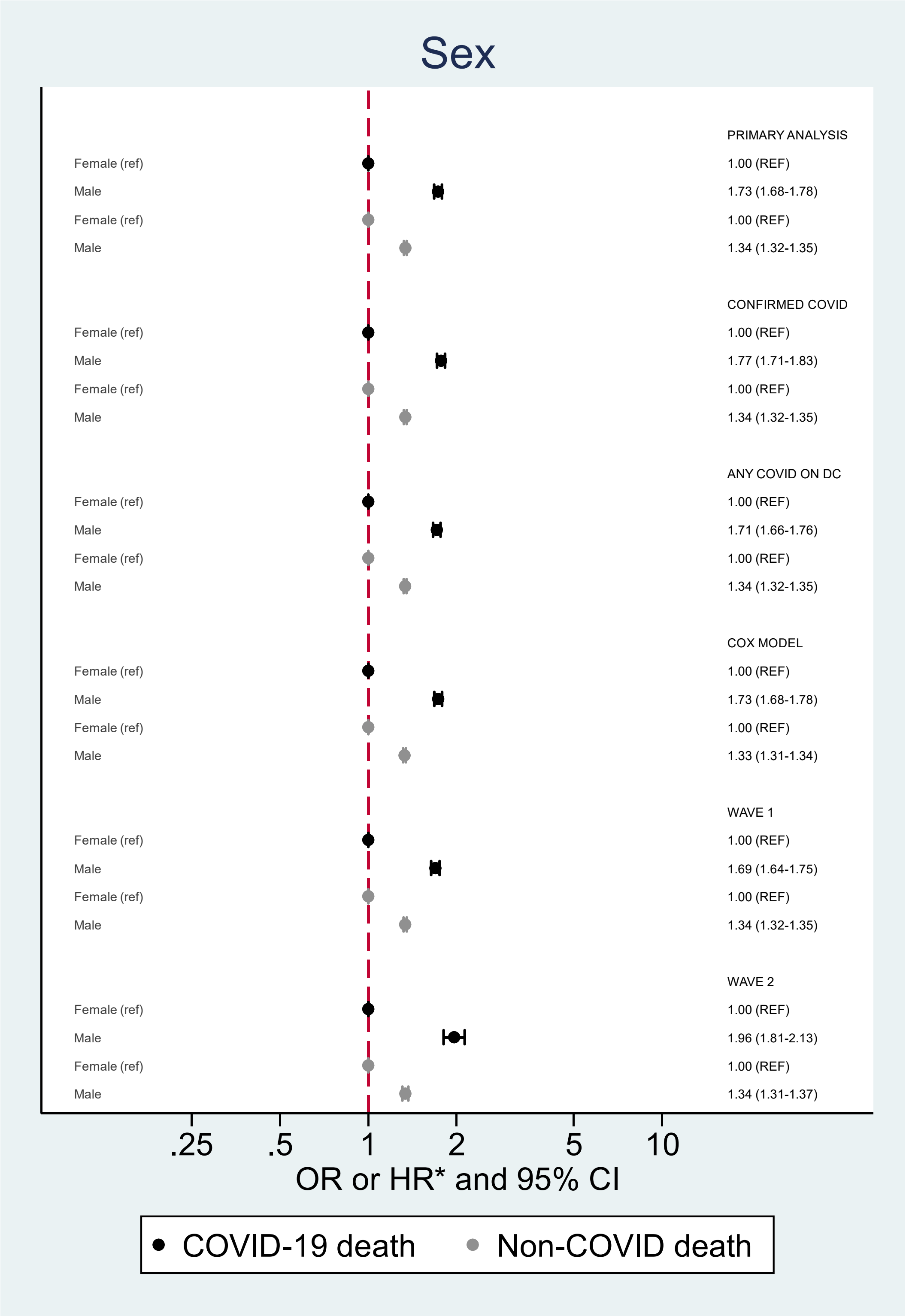

*CONFIRMED COVID = COVID-19 outcome defined using only the ICD-10 code U07.1 (COVID-19 – virus identified); ANY COVID ON DC = COVID-19 outcome defined as a COVID-19 code anywhere on the death certificate, rather than only as the underlying cause of death; COX MODEL = Hazard ratios from a Cox regression model (containing age / sex / covariate of interest and stratified by STP); WAVE 1 = 1st February to 31st August; WAVE 2 = 1st September to 9th November. *HR for the Cox model results, OR for all other results.*

(c)

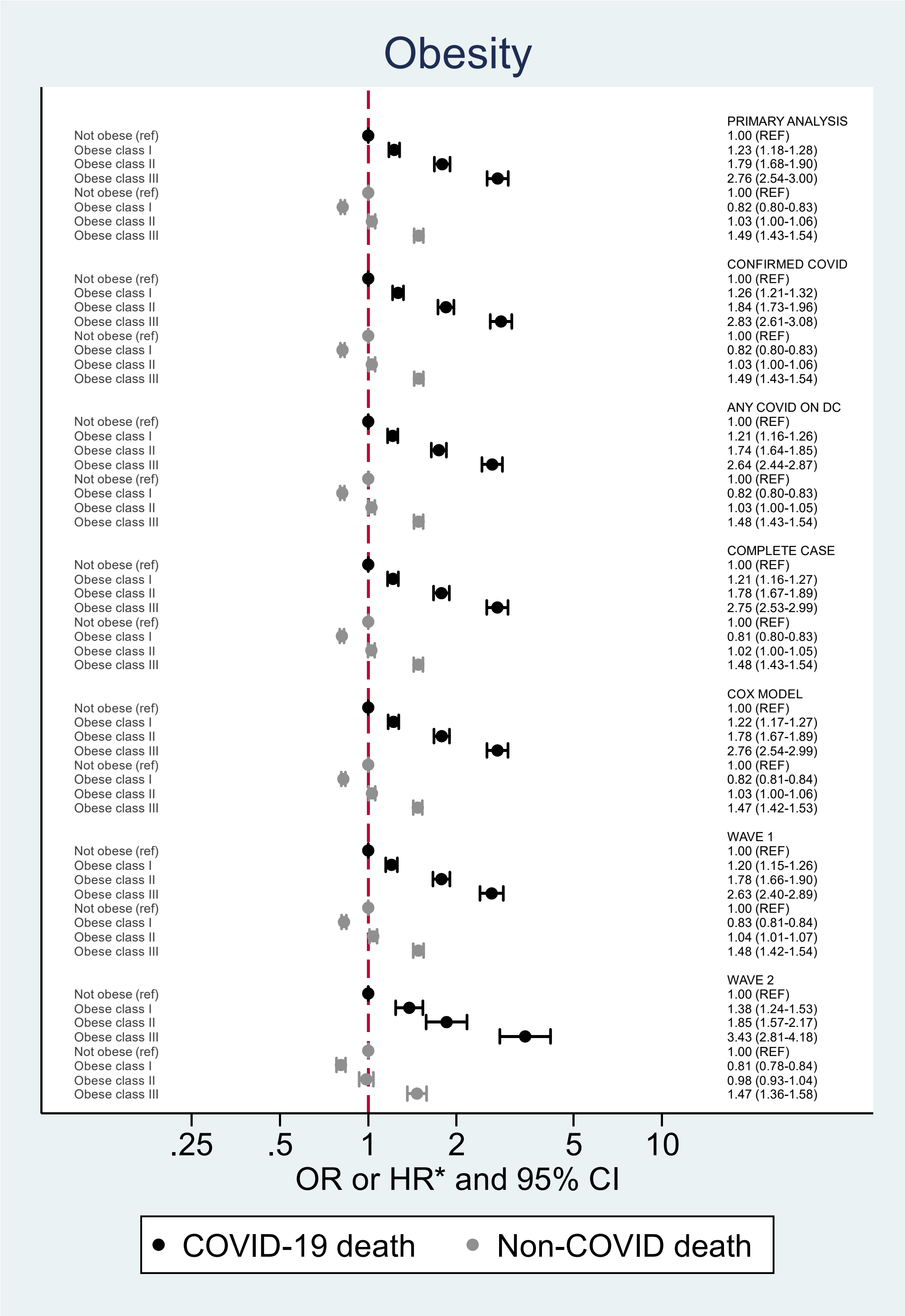

*CONFIRMED COVID = COVID-19 outcome defined using only the ICD-10 code U07.1 (COVID-19 – virus identified); ANY COVID ON DC = COVID-19 outcome defined as a COVID-19 code anywhere on the death certificate, rather than only as the underlying cause of death; COMPLETE CASE = people with missing BMI excluded; COX MODEL = Hazard ratios from a Cox regression model (containing age / sex / covariate of interest and stratified by STP); WAVE 1 = 1st February to 31st August; WAVE 2 = 1st September to 9th November. *HR for the Cox model results, OR for all other results.*

(d)

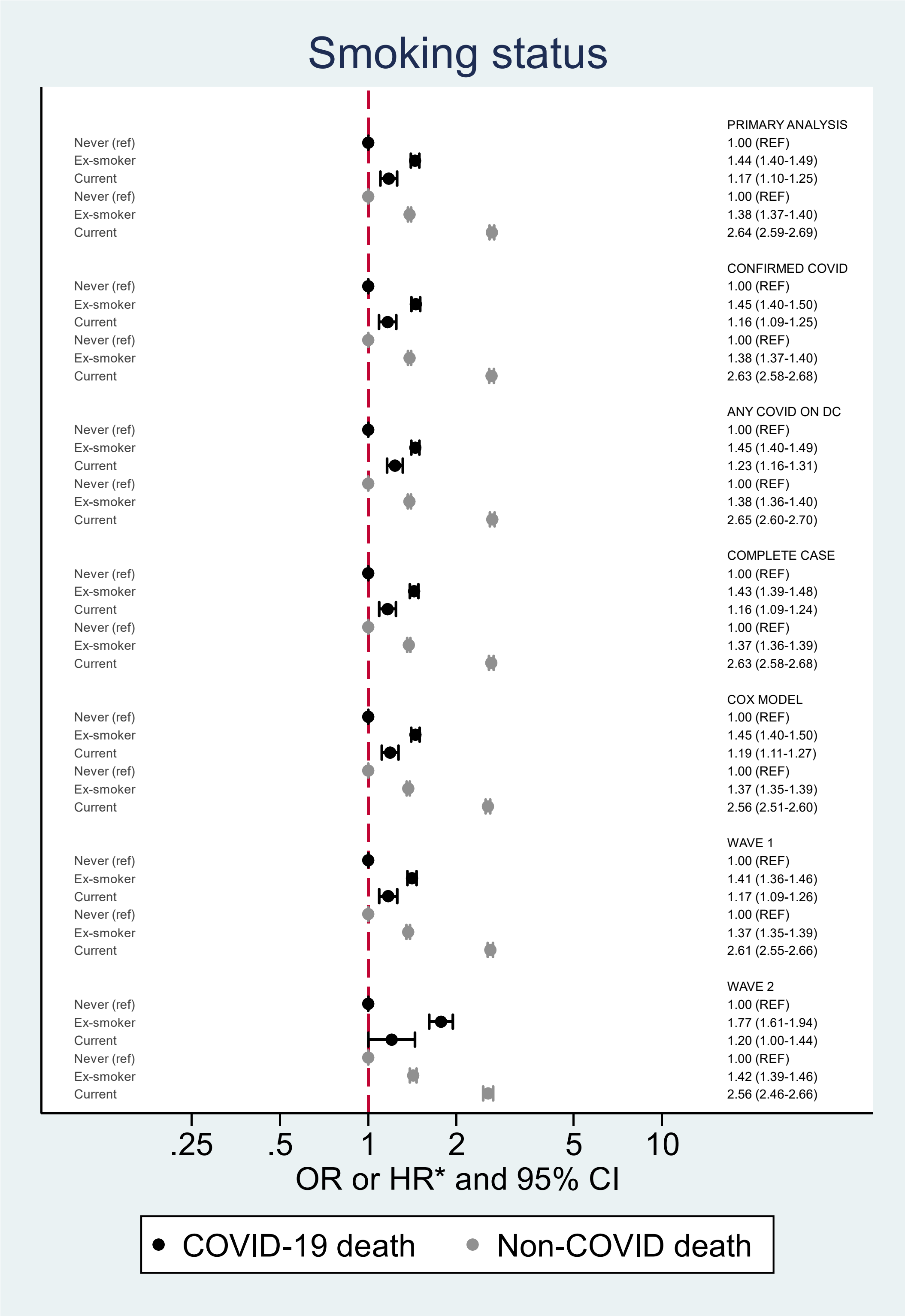

*CONFIRMED COVID = COVID-19 outcome defined using only the ICD-10 code U07.1 (COVID-19 – virus identified); ANY COVID ON DC = COVID-19 outcome defined as a COVID-19 code anywhere on the death certificate, rather than only as the underlying cause of death; COMPLETE CASE = people with missing smoking excluded; COX MODEL = Hazard ratios from a Cox regression model (containing age / sex / covariate of interest and stratified by STP); WAVE 1 = 1st February to 31st August; WAVE 2 = 1st September to 9th November. *HR for the Cox model results, OR for all other results.*

(e)

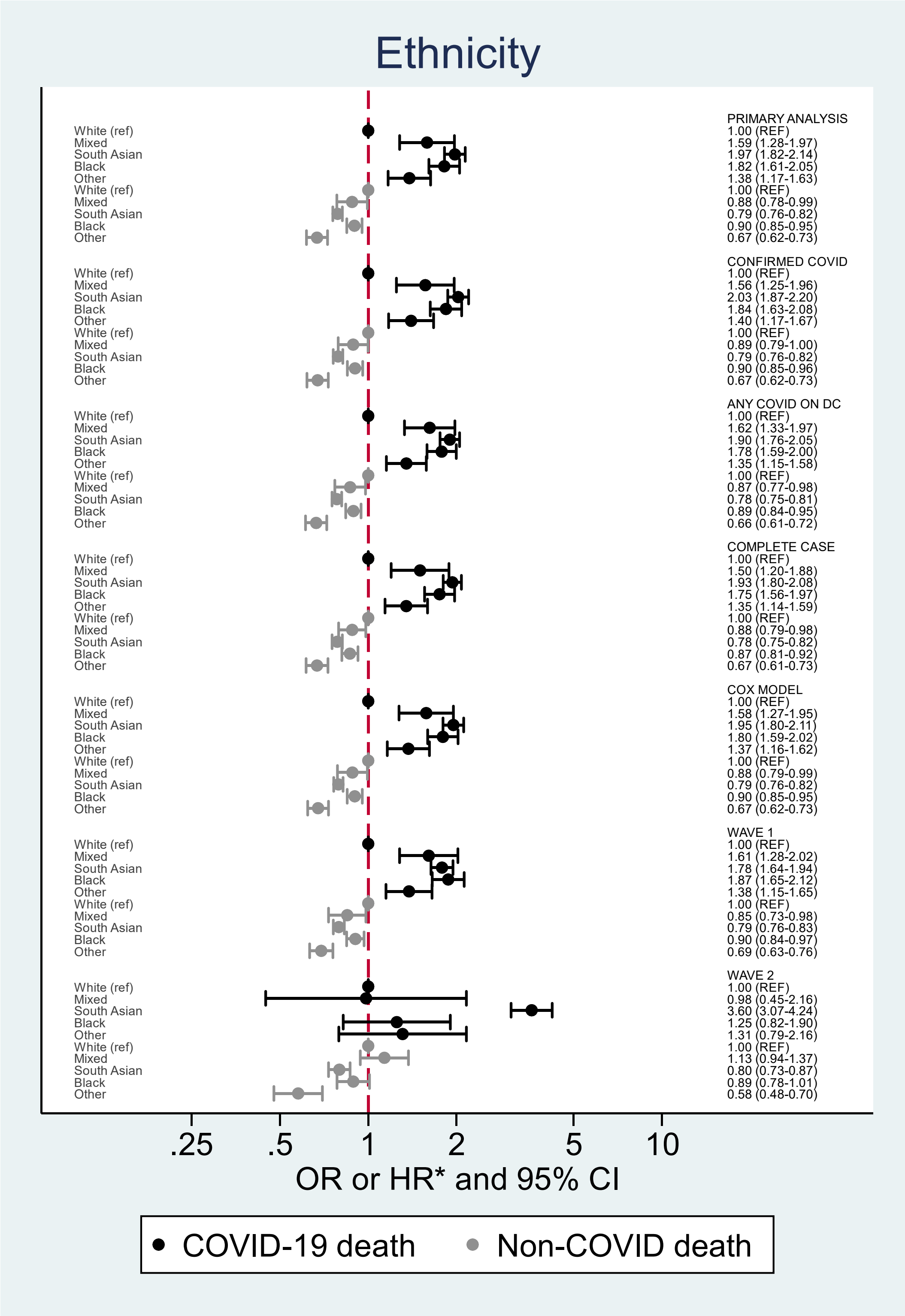

*CONFIRMED COVID = COVID-19 outcome defined using only the ICD-10 code U07.1 (COVID-19 – virus identified); ANY COVID ON DC = COVID-19 outcome defined as a COVID-19 code anywhere on the death certificate, rather than only as the underlying cause of death; COMPLETE CASE = people with missing ethnicity excluded; COX MODEL = Hazard ratios from a Cox regression model (containing age / sex / covariate of interest and stratified by STP); WAVE 1 = 1st February to 31st August; WAVE 2 = 1st September to 9th November. *HR for the Cox model results, OR for all other results.*

(f)

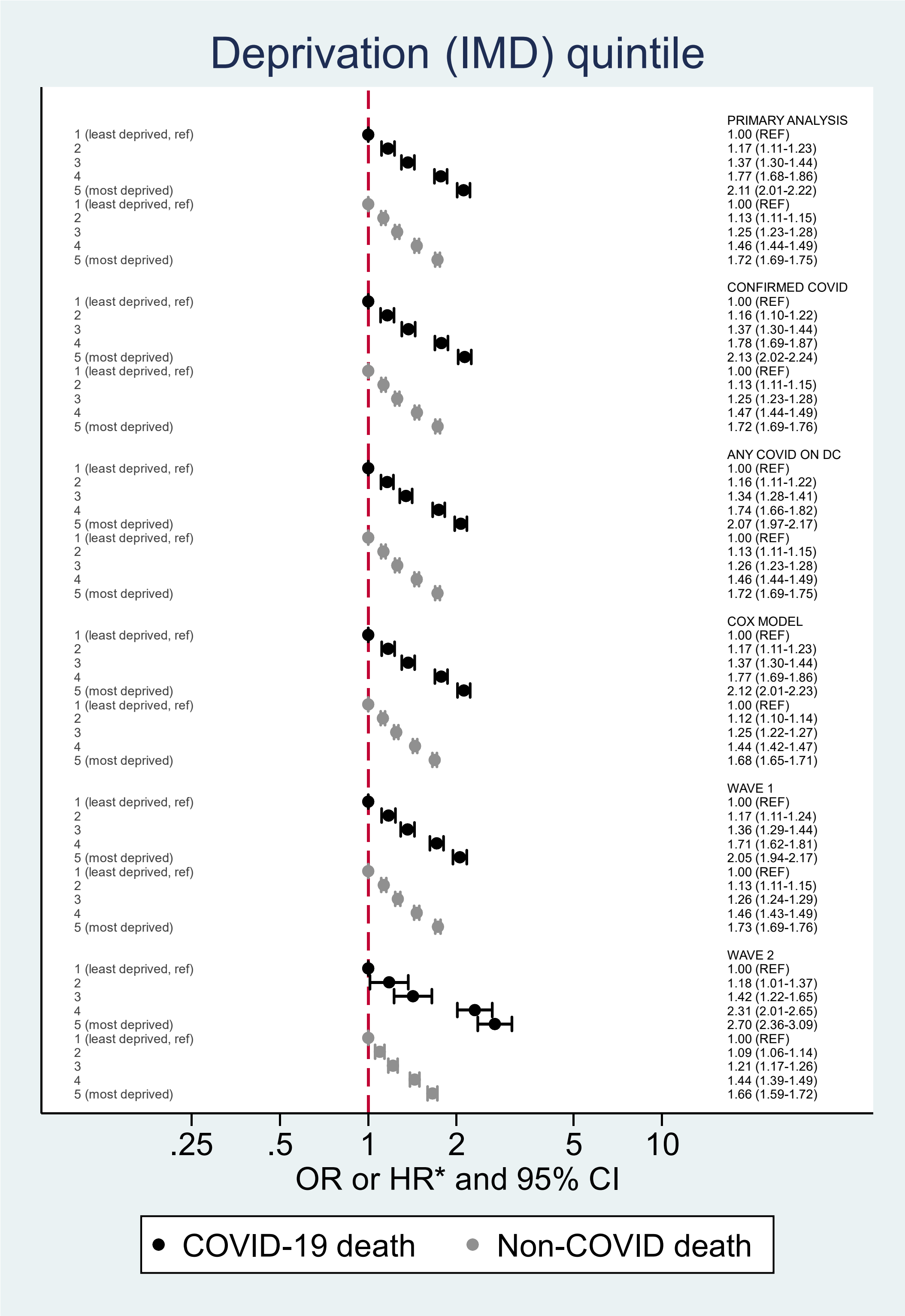

*CONFIRMED COVID = COVID-19 outcome defined using only the ICD-10 code U07.1 (COVID-19 – virus identified); ANY COVID ON DC = COVID-19 outcome defined as a COVID-19 code anywhere on the death certificate, rather than only as the underlying cause of death; COX MODEL = Hazard ratios from a Cox regression model (containing age / sex / covariate of interest and stratified by STP); WAVE 1 = 1st February to 31st August; WAVE 2 = 1st September to 9th November. *HR for the Cox model results, OR for all other results.*

(g)

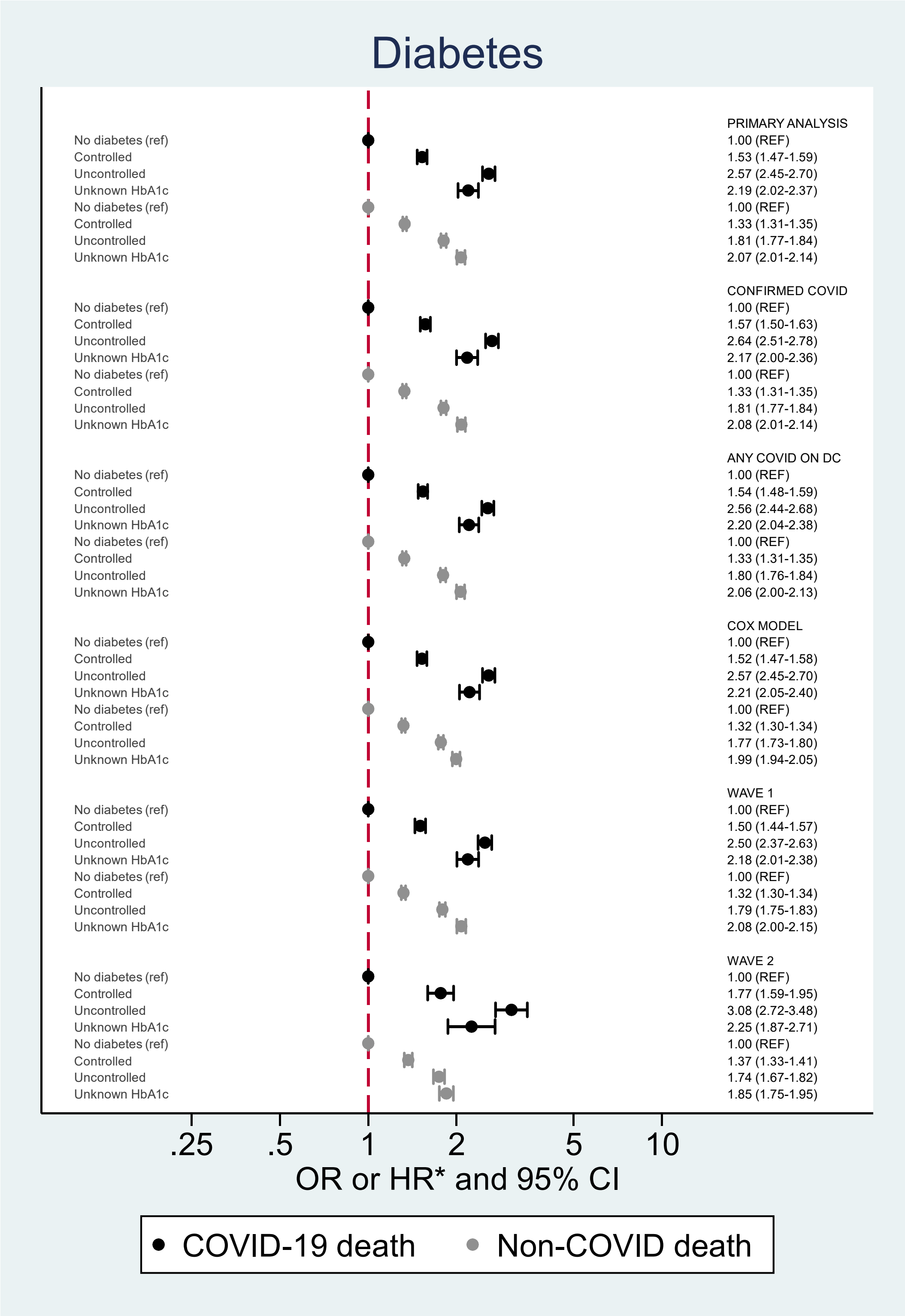

*CONFIRMED COVID = COVID-19 outcome defined using only the ICD-10 code U07.1 (COVID-19 – virus identified); ANY COVID ON DC = COVID-19 outcome defined as a COVID-19 code anywhere on the death certificate, rather than only as the underlying cause of death; COX MODEL = Hazard ratios from a Cox regression model (containing age / sex / covariate of interest and stratified by STP); WAVE 1 = 1st February to 31st August; WAVE 2 = 1st September to 9th November. *HR for the Cox model results, OR for all other results.*

(h)

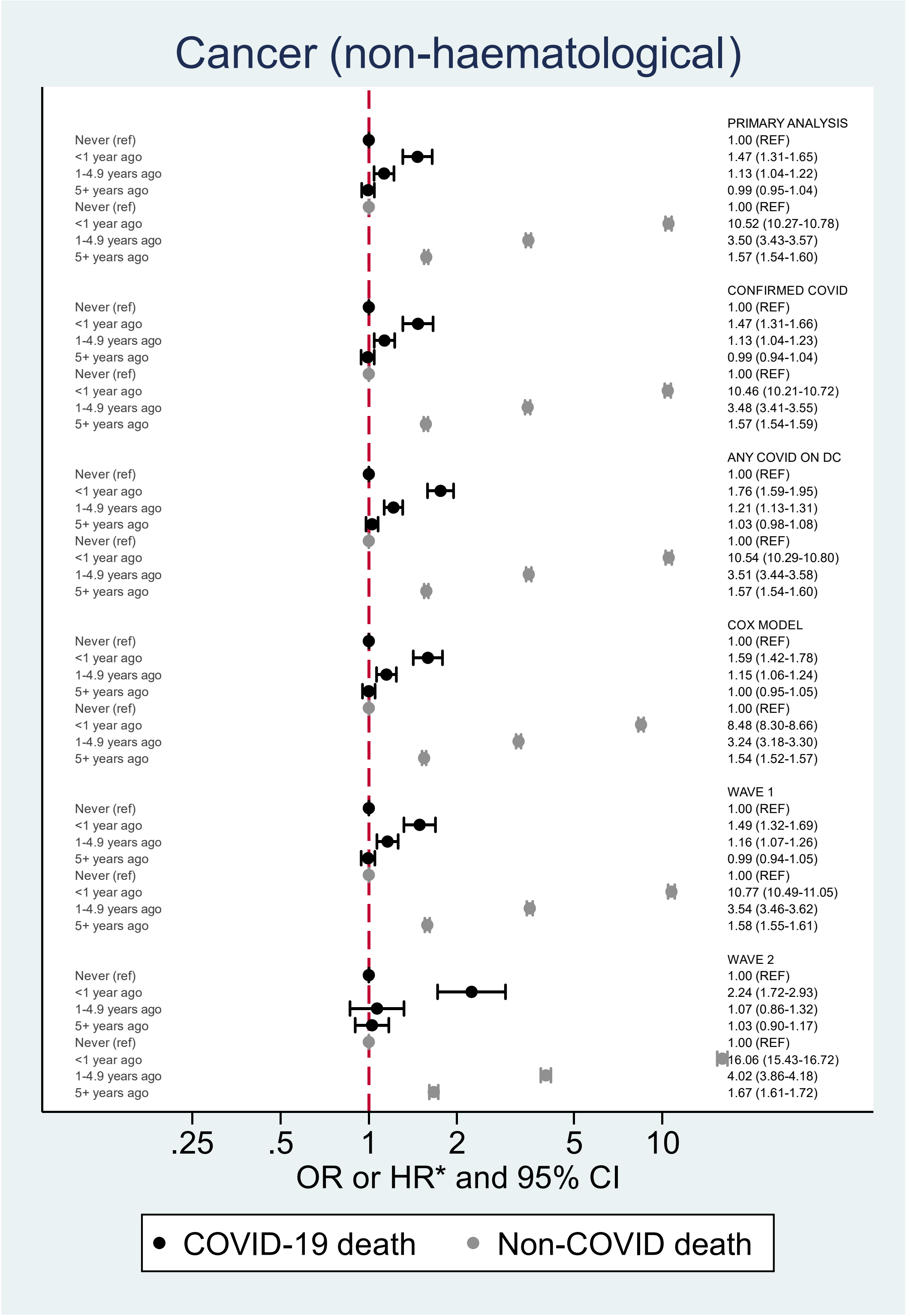

*CONFIRMED COVID = COVID-19 outcome defined using only the ICD-10 code U07.1 (COVID-19 – virus identified); ANY COVID ON DC = COVID-19 outcome defined as a COVID-19 code anywhere on the death certificate, rather than only as the underlying cause of death; COX MODEL = Hazard ratios from a Cox regression model (containing age / sex / covariate of interest and stratified by STP); WAVE 1 = 1st February to 31st August; WAVE 2 = 1st September to 9th November. *HR for the Cox model results, OR for all other results.*

(i)

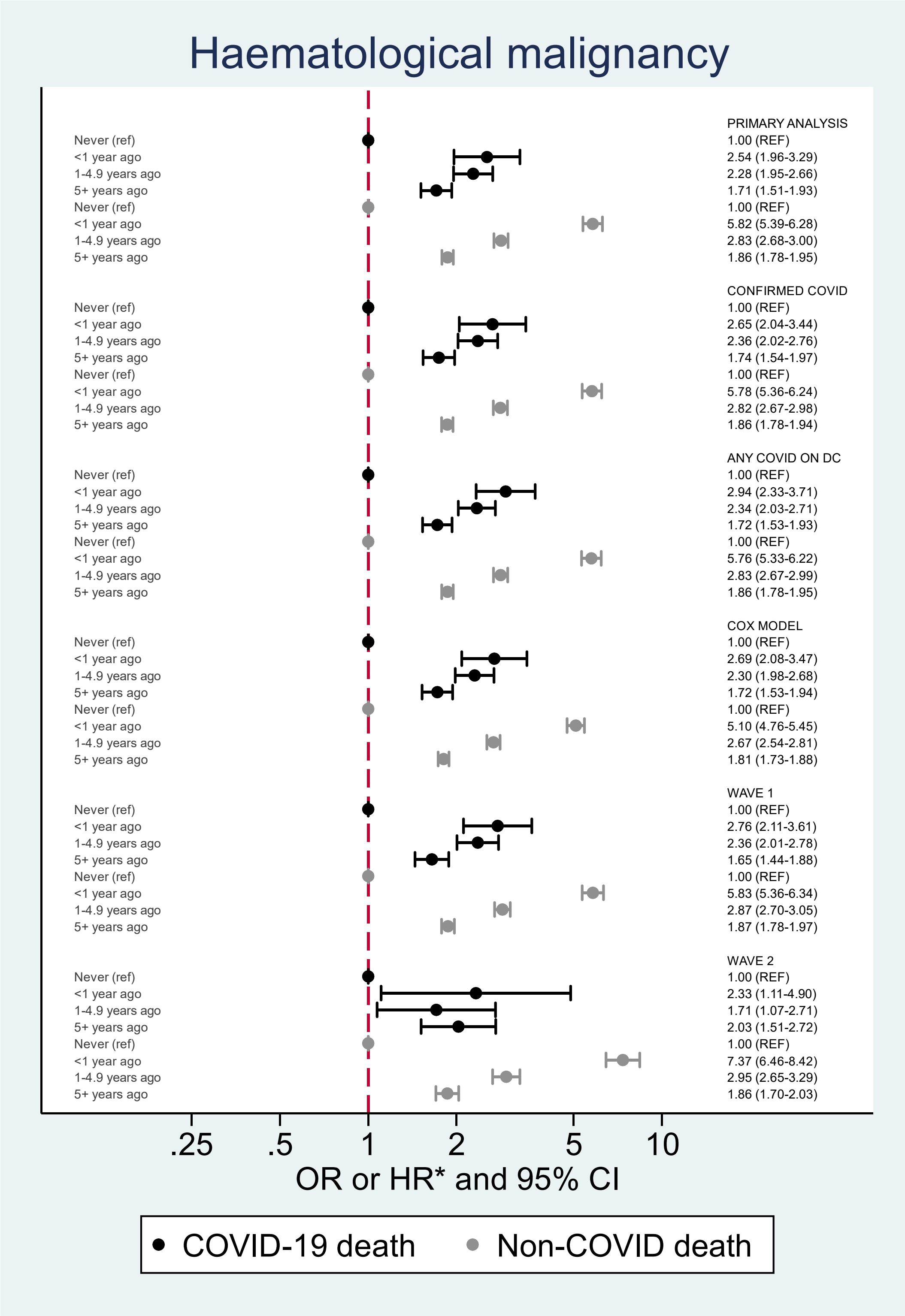

*CONFIRMED COVID = COVID-19 outcome defined using only the ICD-10 code U07.1 (COVID-19 – virus identified); ANY COVID ON DC = COVID-19 outcome defined as a COVID-19 code anywhere on the death certificate, rather than only as the underlying cause of death; COX MODEL = Hazard ratios from a Cox regression model (containing age / sex / covariate of interest and stratified by STP); WAVE 1 = 1st February to 31st August; WAVE 2 = 1st September to 9th November. *HR for the Cox model results, OR for all other results.*

(j)

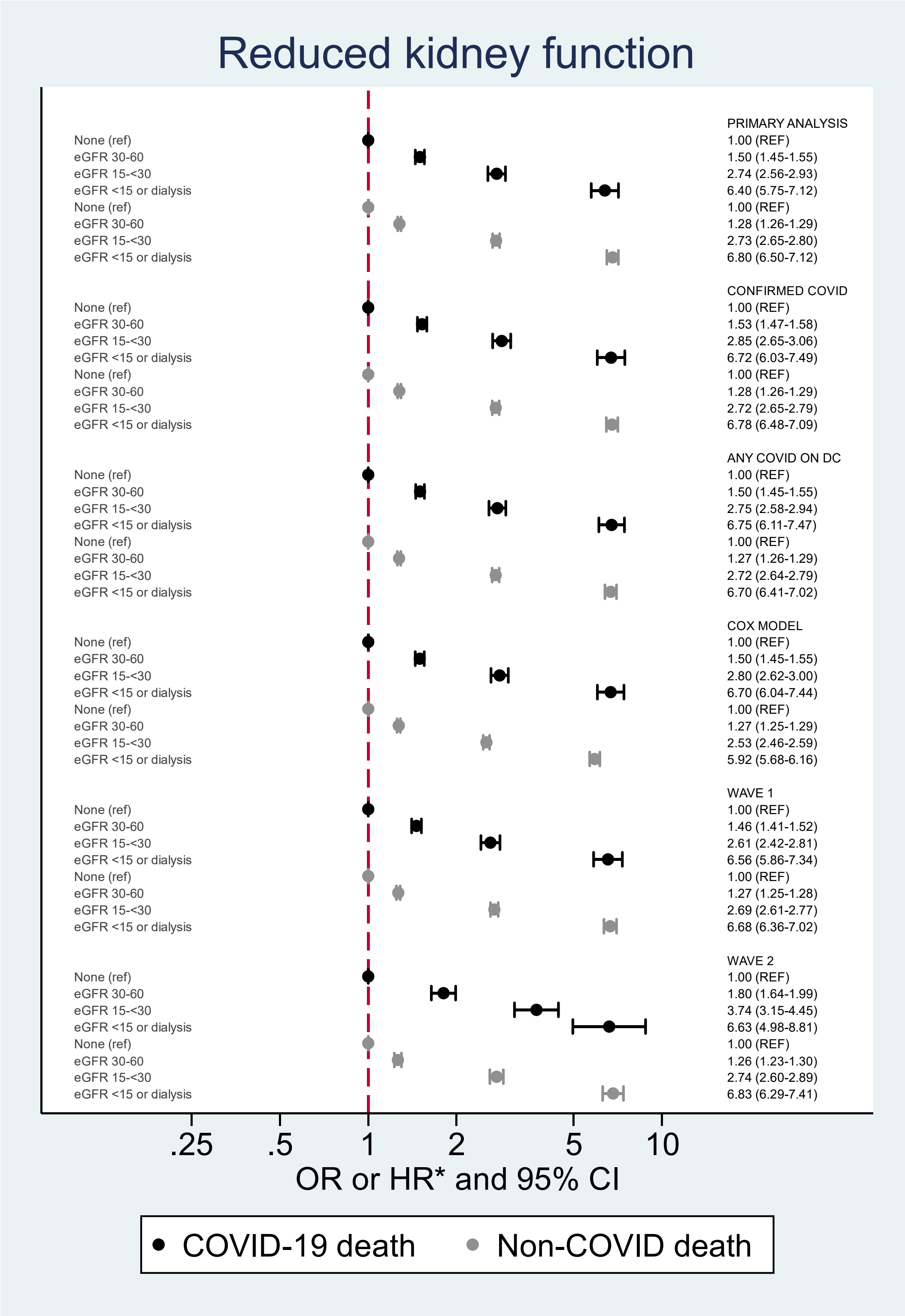

*CONFIRMED COVID = COVID-19 outcome defined using only the ICD-10 code U07.1 (COVID-19 – virus identified); ANY COVID ON DC = COVID-19 outcome defined as a COVID-19 code anywhere on the death certificate, rather than only as the underlying cause of death; COX MODEL = Hazard ratios from a Cox regression model (containing age / sex / covariate of interest and stratified by STP); WAVE 1 = 1st February to 31st August; WAVE 2 = 1st September to 9th November. *HR for the Cox model results, OR for all other results.*

(k)

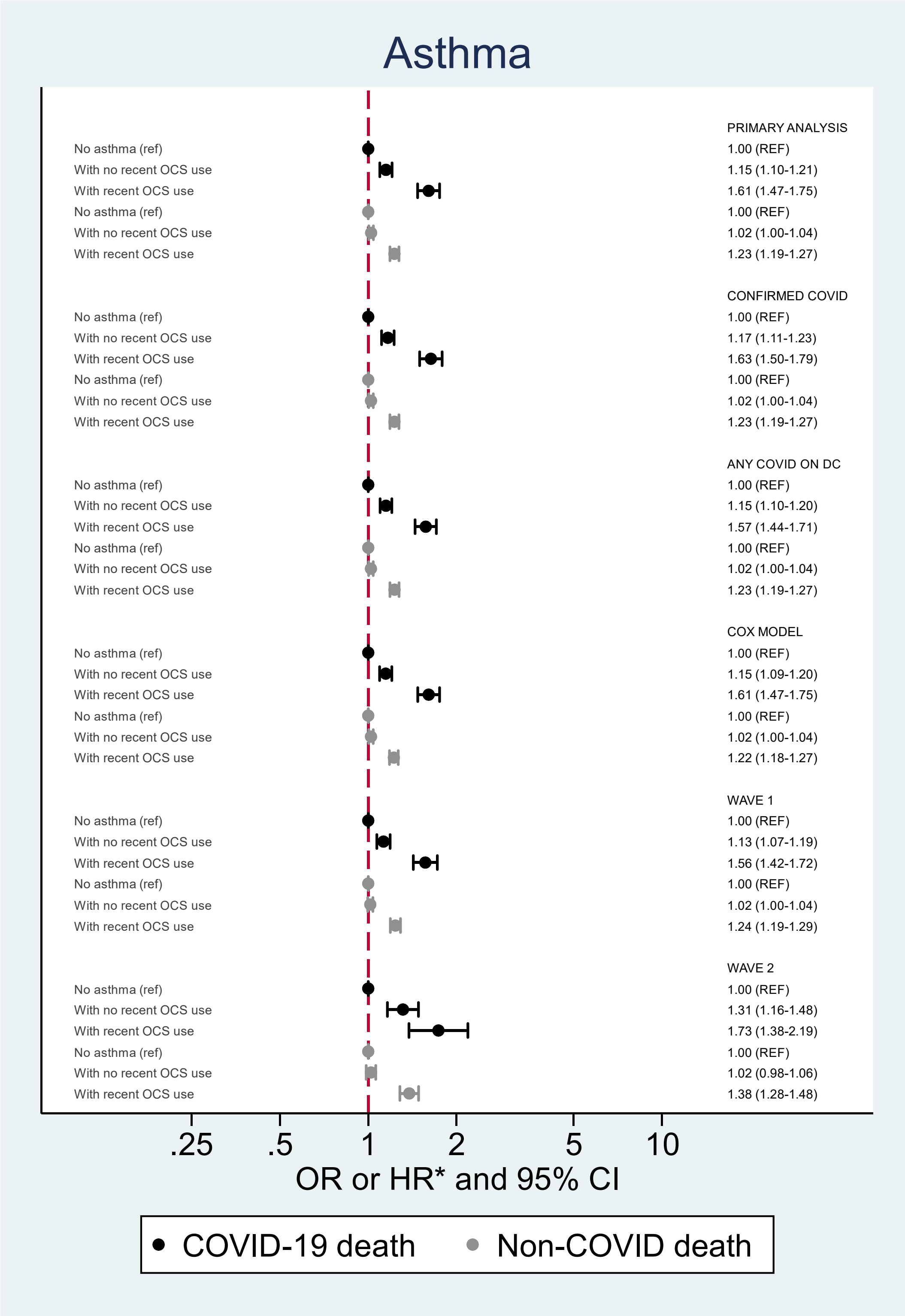

*CONFIRMED COVID = COVID-19 outcome defined using only the ICD-10 code U07.1 (COVID-19 – virus identified); ANY COVID ON DC = COVID-19 outcome defined as a COVID-19 code anywhere on the death certificate, rather than only as the underlying cause of death; COX MODEL = Hazard ratios from a Cox regression model (containing age / sex / covariate of interest and stratified by STP); WAVE 1 = 1st February to 31st August; WAVE 2 = 1st September to 9th November. *HR for the Cox model results, OR for all other results.*

(l)

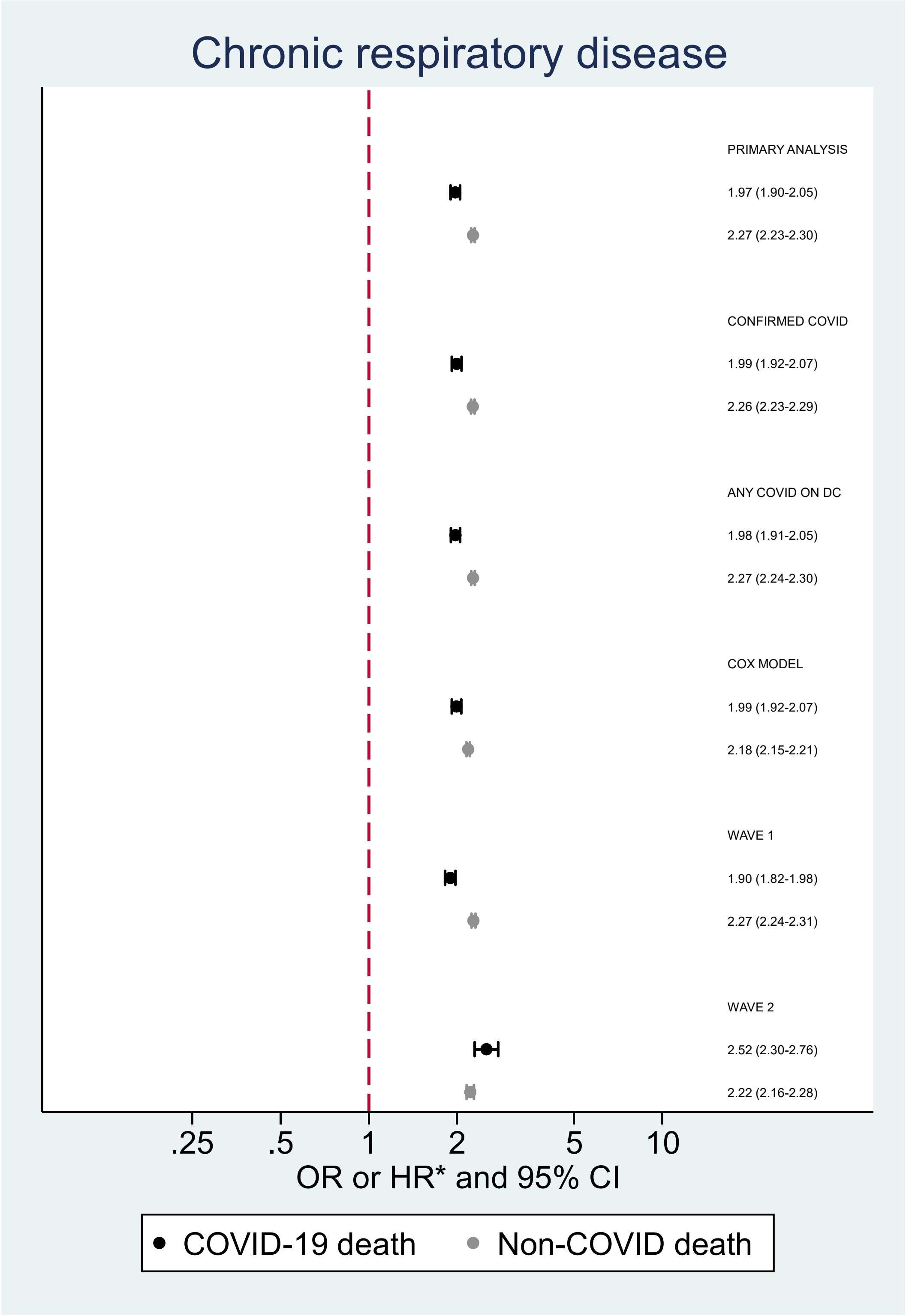

*CONFIRMED COVID = COVID-19 outcome defined using only the ICD-10 code U07.1 (COVID-19 – virus identified); ANY COVID ON DC = COVID-19 outcome defined as a COVID-19 code anywhere on the death certificate, rather than only as the underlying cause of death; COX MODEL = Hazard ratios from a Cox regression model (containing age / sex / covariate of interest and stratified by STP); WAVE 1 = 1st February to 31st August; WAVE 2 = 1st September to 9th November. *HR for the Cox model results, OR for all other results.*

(m)

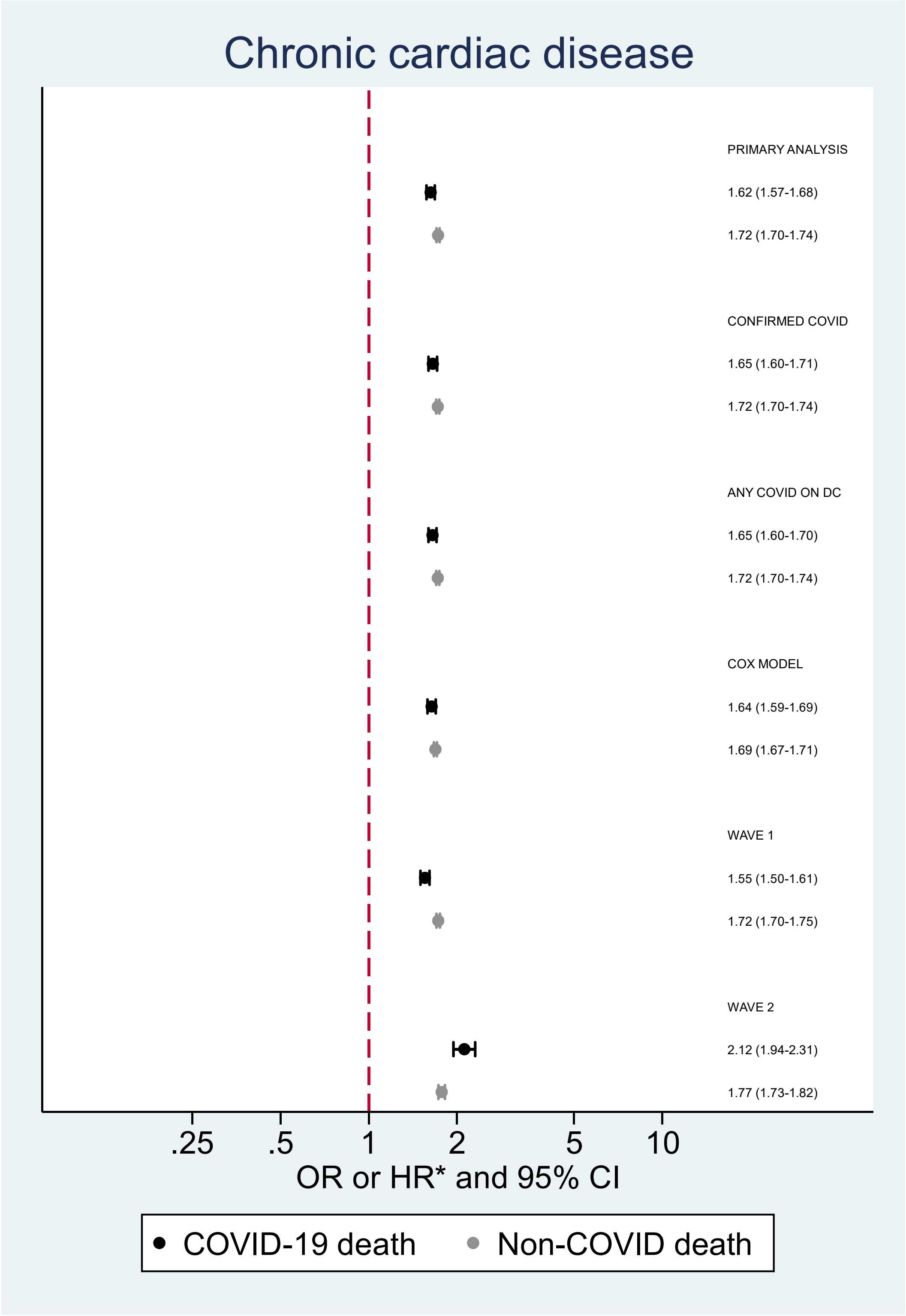

*CONFIRMED COVID = COVID-19 outcome defined using only the ICD-10 code U07.1 (COVID-19 – virus identified); ANY COVID ON DC = COVID-19 outcome defined as a COVID-19 code anywhere on the death certificate, rather than only as the underlying cause of death; COX MODEL = Hazard ratios from a Cox regression model (containing age / sex / covariate of interest and stratified by STP); WAVE 1 = 1st February to 31st August; WAVE 2 = 1st September to 9th November. *HR for the Cox model results, OR for all other results.*

(n)

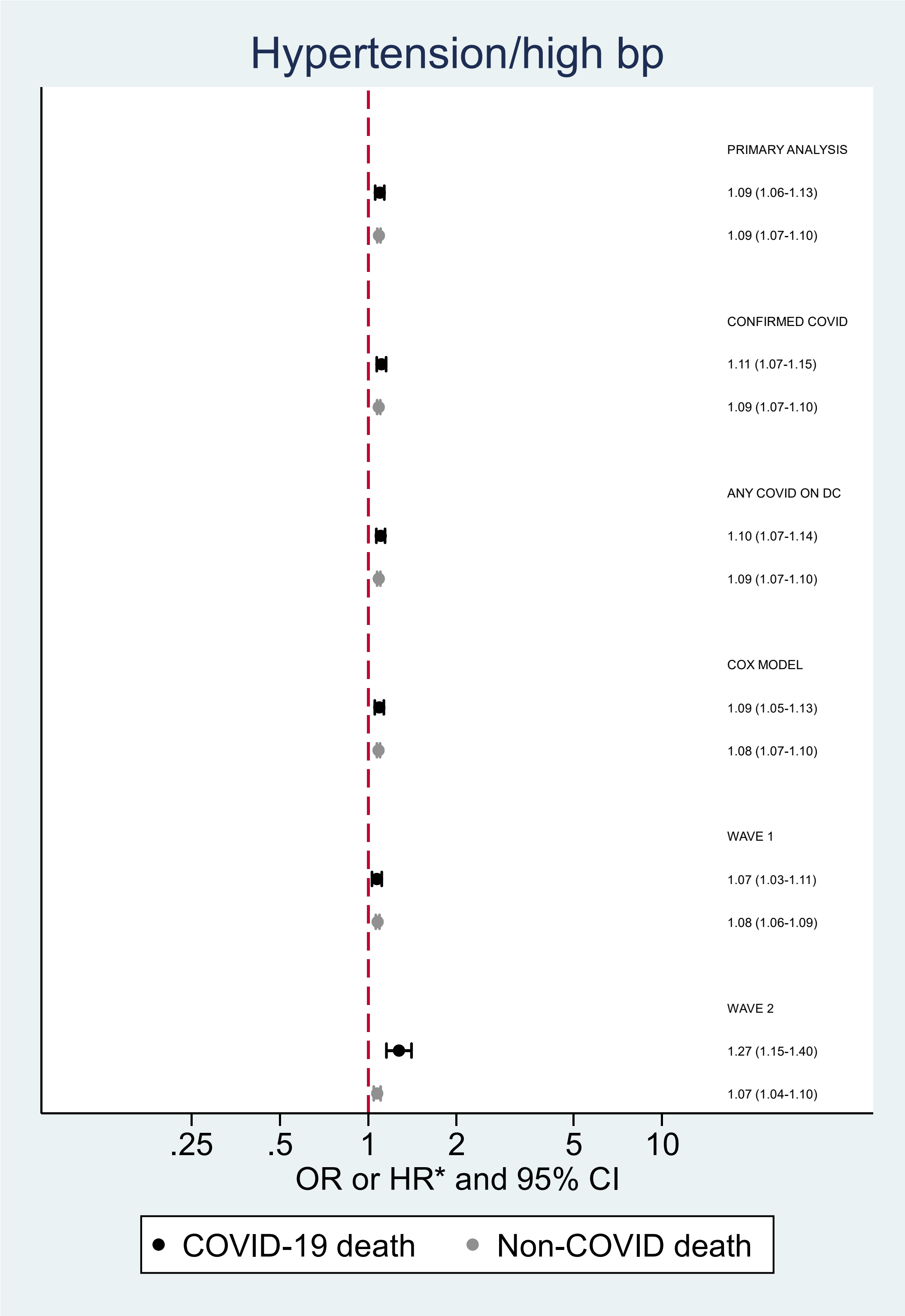

*CONFIRMED COVID = COVID-19 outcome defined using only the ICD-10 code U07.1 (COVID-19 – virus identified); ANY COVID ON DC = COVID-19 outcome defined as a COVID-19 code anywhere on the death certificate, rather than only as the underlying cause of death; COX MODEL = Hazard ratios from a Cox regression model (containing age / sex / covariate of interest and stratified by STP); WAVE 1 = 1st February to 31st August; WAVE 2 = 1st September to 9th November. *HR for the Cox model results, OR for all other results.*

(o)

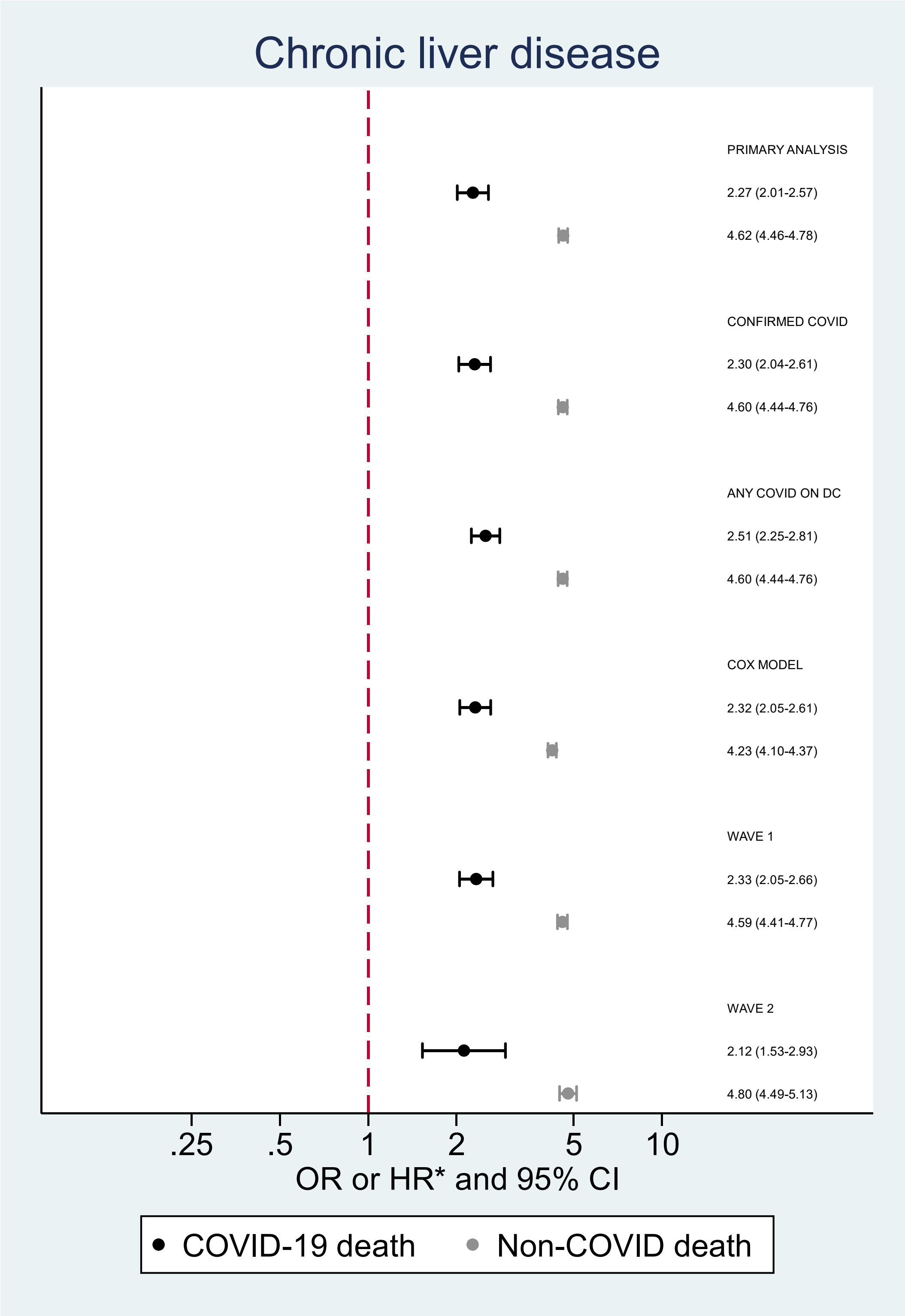

*CONFIRMED COVID = COVID-19 outcome defined using only the ICD-10 code U07.1 (COVID-19 – virus identified); ANY COVID ON DC = COVID-19 outcome defined as a COVID-19 code anywhere on the death certificate, rather than only as the underlying cause of death; COX MODEL = Hazard ratios from a Cox regression model (containing age / sex / covariate of interest and stratified by STP); WAVE 1 = 1st February to 31st August; WAVE 2 = 1st September to 9th November. *HR for the Cox model results, OR for all other results.*

(p)

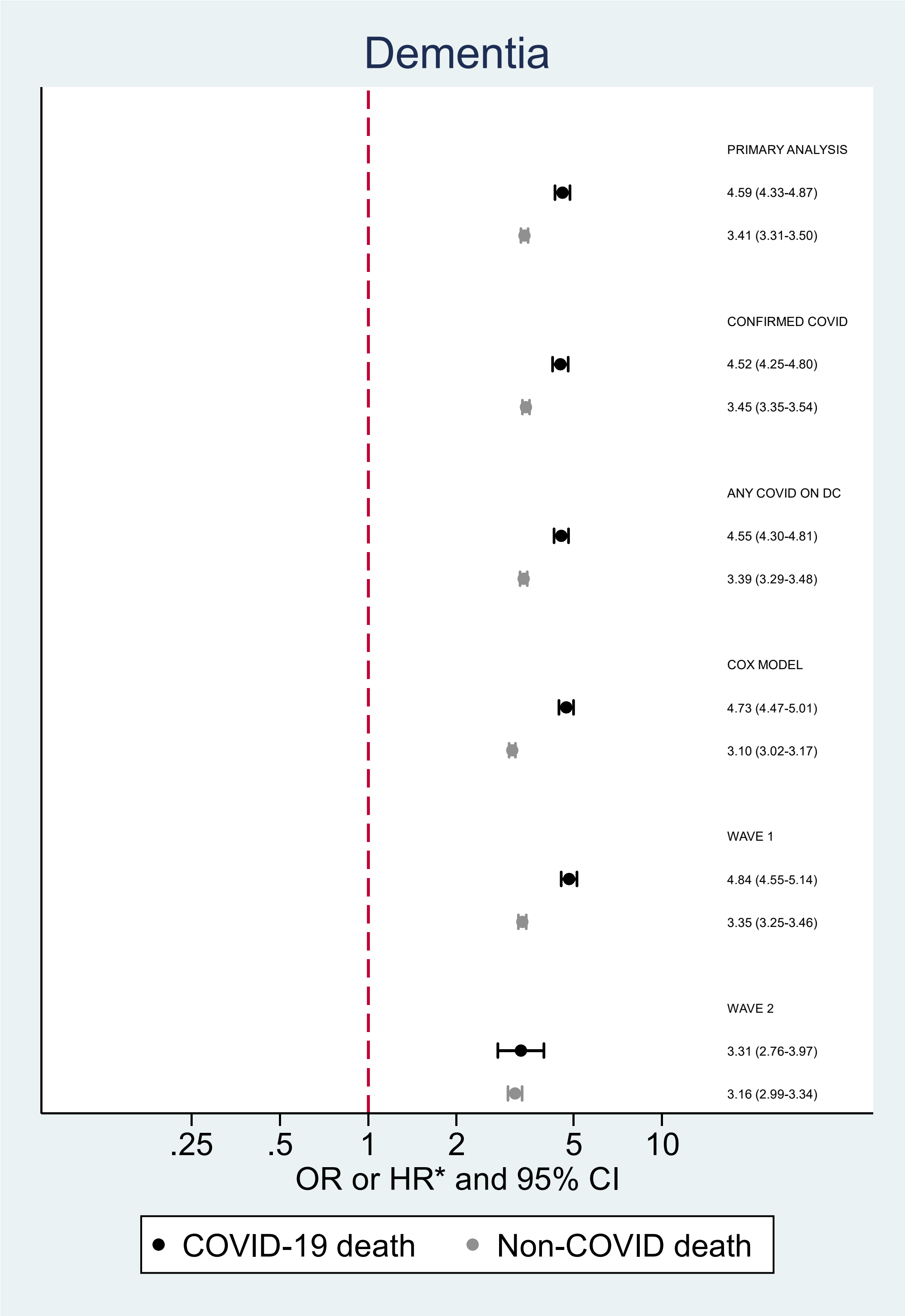

*CONFIRMED COVID = COVID-19 outcome defined using only the ICD-10 code U07.1 (COVID-19 – virus identified); ANY COVID ON DC = COVID-19 outcome defined as a COVID-19 code anywhere on the death certificate, rather than only as the underlying cause of death; COX MODEL = Hazard ratios from a Cox regression model (containing age / sex / covariate of interest and stratified by STP); WAVE 1 = 1st February to 31st August; WAVE 2 = 1st September to 9th November. *HR for the Cox model results, OR for all other results.*

(q)

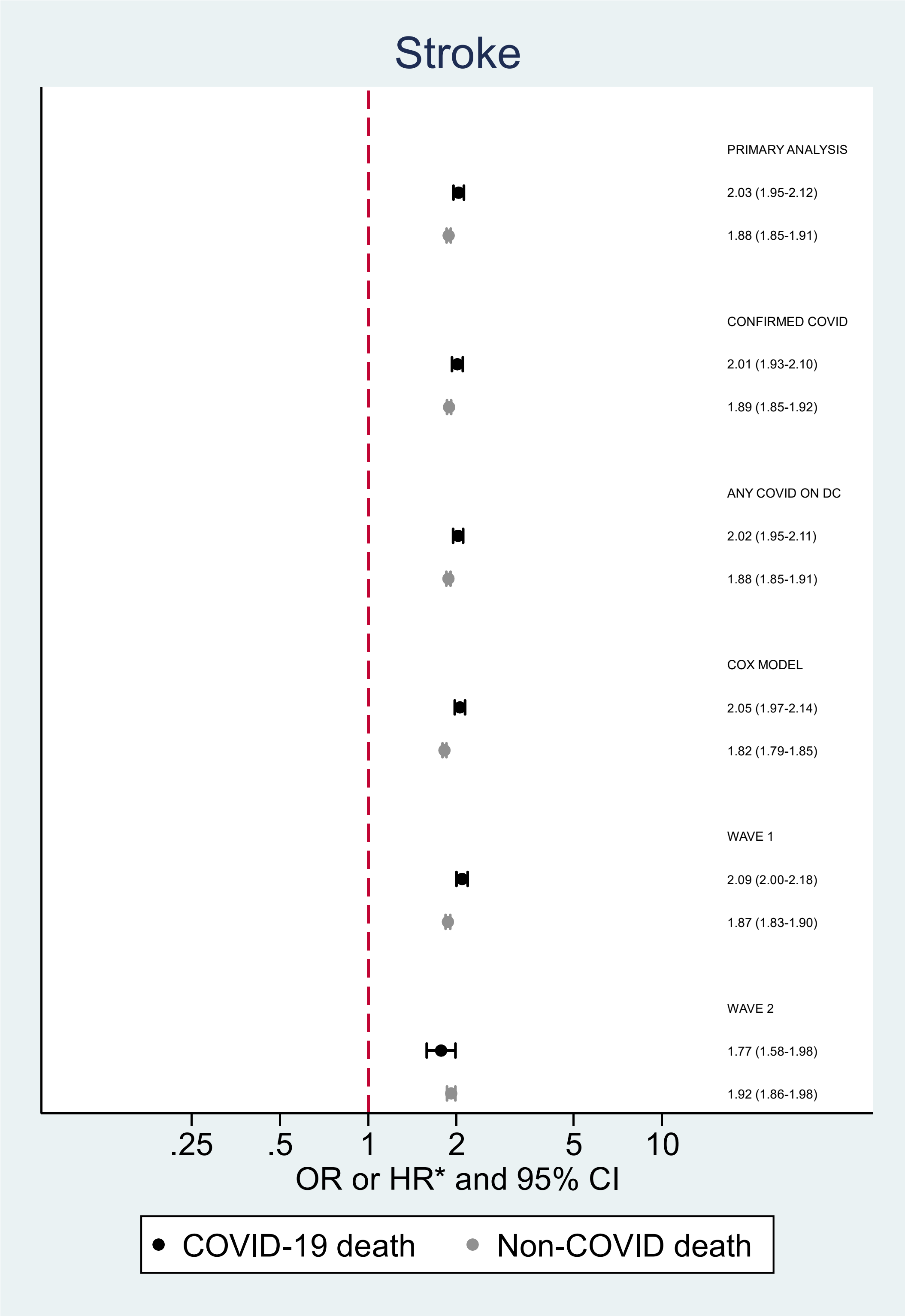

*CONFIRMED COVID = COVID-19 outcome defined using only the ICD-10 code U07.1 (COVID-19 – virus identified); ANY COVID ON DC = COVID-19 outcome defined as a COVID-19 code anywhere on the death certificate, rather than only as the underlying cause of death; COX MODEL = Hazard ratios from a Cox regression model (containing age / sex / covariate of interest and stratified by STP); WAVE 1 = 1st February to 31st August; WAVE 2 = 1st September to 9th November. *HR for the Cox model results, OR for all other results.*

(r)

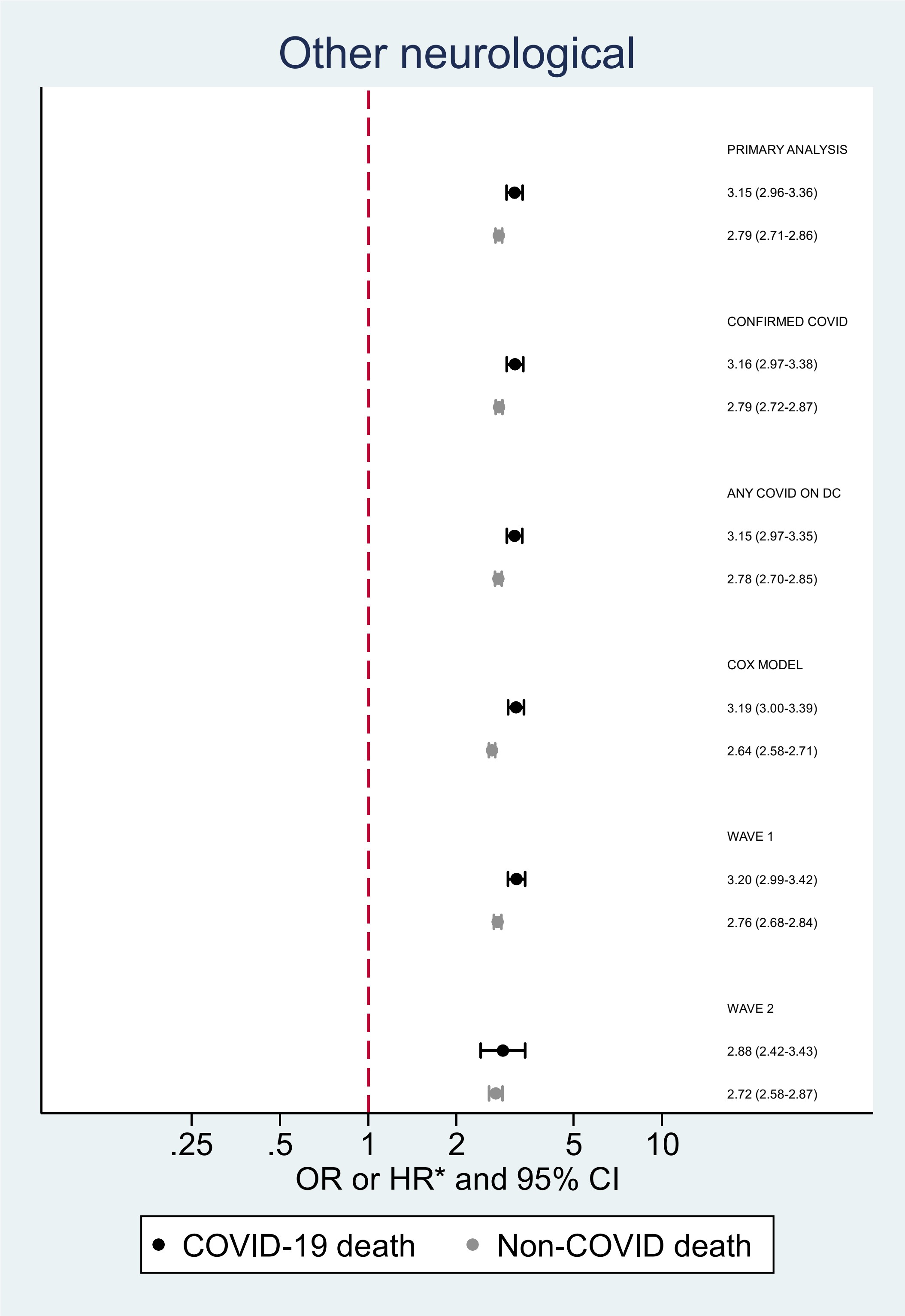

*CONFIRMED COVID = COVID-19 outcome defined using only the ICD-10 code U07.1 (COVID-19 – virus identified); ANY COVID ON DC = COVID-19 outcome defined as a COVID-19 code anywhere on the death certificate, rather than only as the underlying cause of death; COX MODEL = Hazard ratios from a Cox regression model (containing age / sex / covariate of interest and stratified by STP); WAVE 1 = 1st February to 31st August; WAVE 2 = 1st September to 9th November. *HR for the Cox model results, OR for all other results.*

(s)

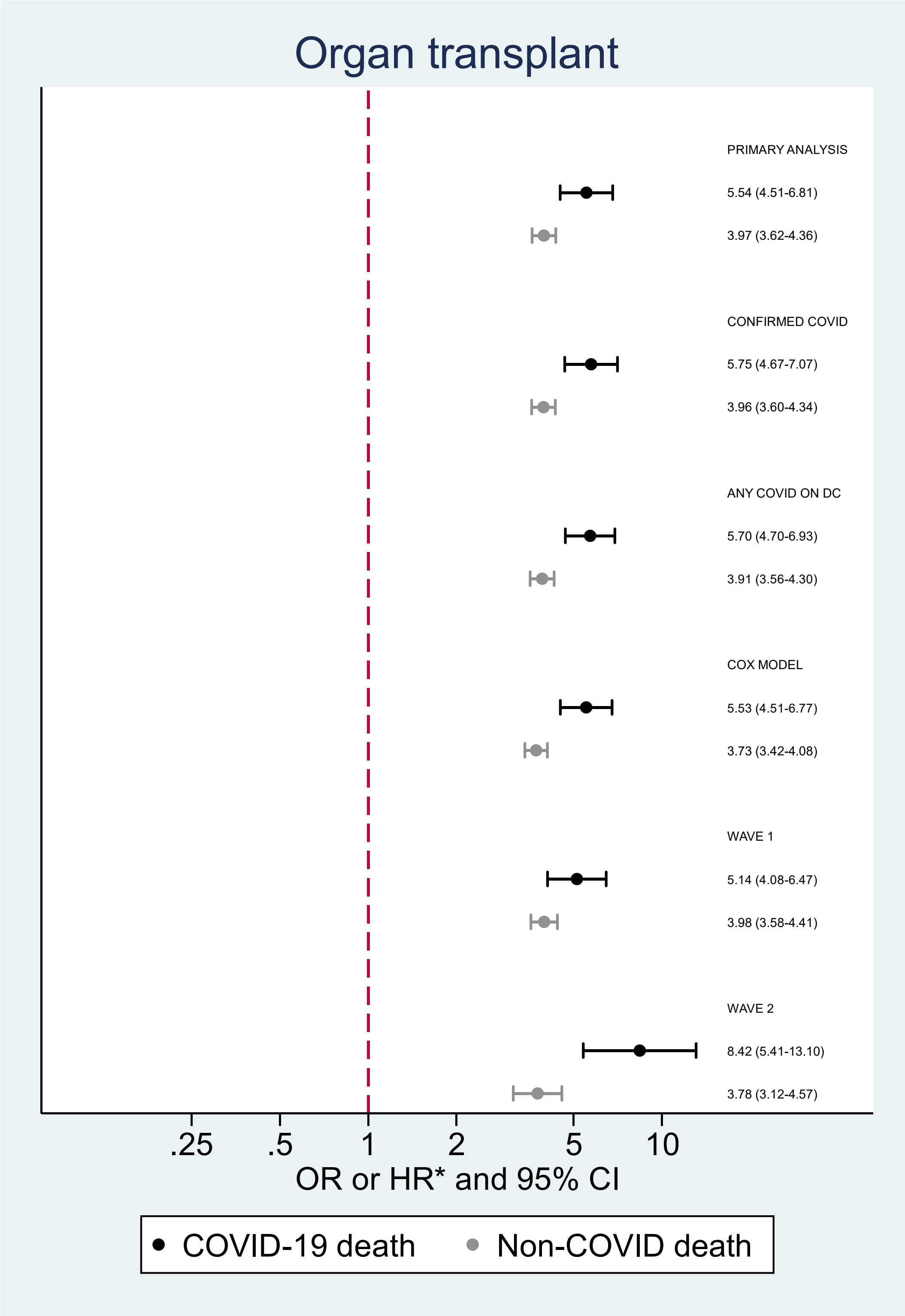

*CONFIRMED COVID = COVID-19 outcome defined using only the ICD-10 code U07.1 (COVID-19 – virus identified); ANY COVID ON DC = COVID-19 outcome defined as a COVID-19 code anywhere on the death certificate, rather than only as the underlying cause of death; COX MODEL = Hazard ratios from a Cox regression model (containing age / sex / covariate of interest and stratified by STP); WAVE 1 = 1st February to 31st August; WAVE 2 = 1st September to 9th November. *HR for the Cox model results, OR for all other results.*

(t)

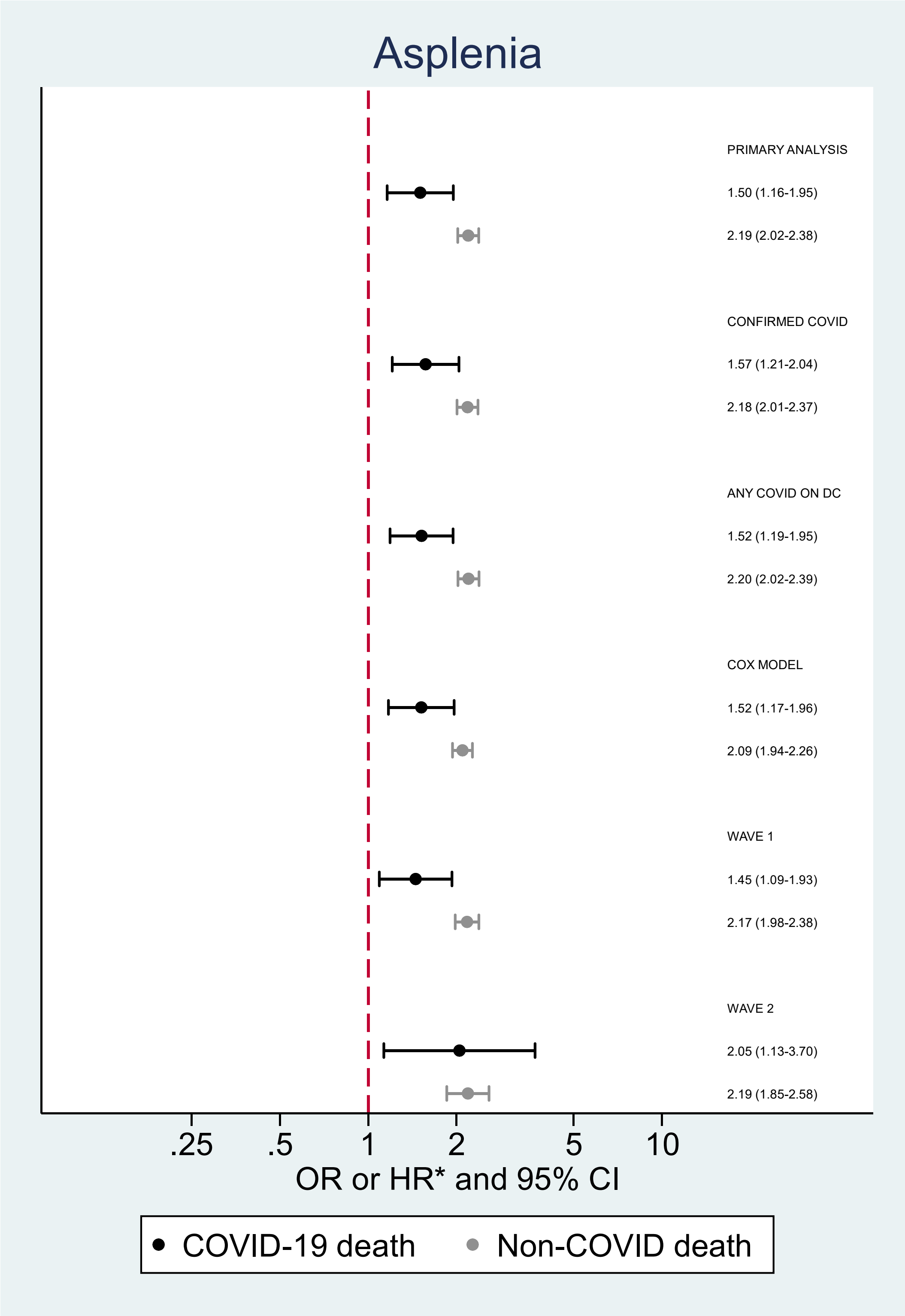

*CONFIRMED COVID = COVID-19 outcome defined using only the ICD-10 code U07.1 (COVID-19 – virus identified); ANY COVID ON DC = COVID-19 outcome defined as a COVID-19 code anywhere on the death certificate, rather than only as the underlying cause of death; COX MODEL = Hazard ratios from a Cox regression model (containing age / sex / covariate of interest and stratified by STP); WAVE 1 = 1st February to 31st August; WAVE 2 = 1st September to 9th November. *HR for the Cox model results, OR for all other results.*

(u)

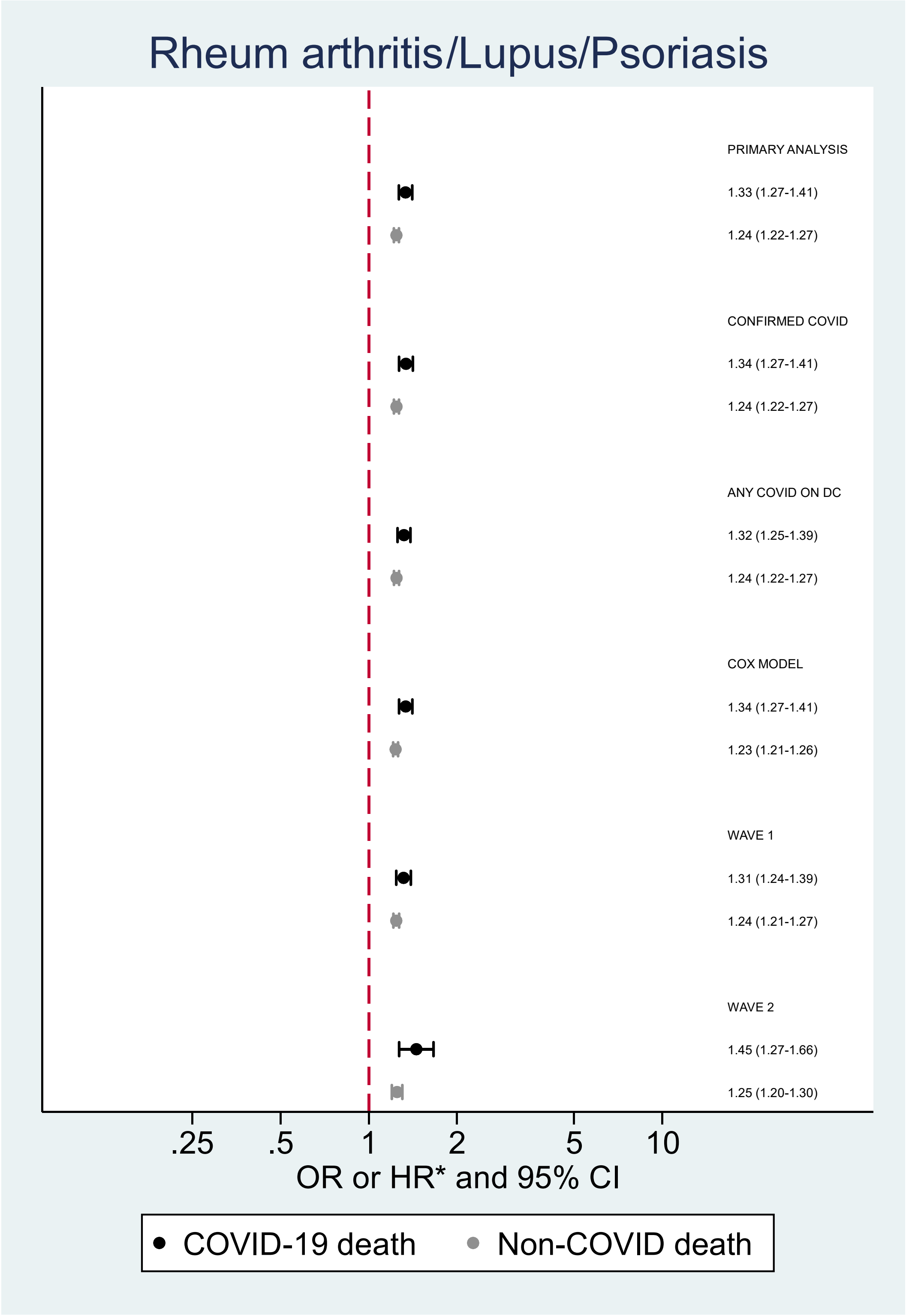

*CONFIRMED COVID = COVID-19 outcome defined using only the ICD-10 code U07.1 (COVID-19 – virus identified); ANY COVID ON DC = COVID-19 outcome defined as a COVID-19 code anywhere on the death certificate, rather than only as the underlying cause of death; COX MODEL = Hazard ratios from a Cox regression model (containing age / sex / covariate of interest and stratified by STP); WAVE 1 = 1st February to 31st August; WAVE 2 = 1st September to 9th November. *HR for the Cox model results, OR for all other results.*

(v)

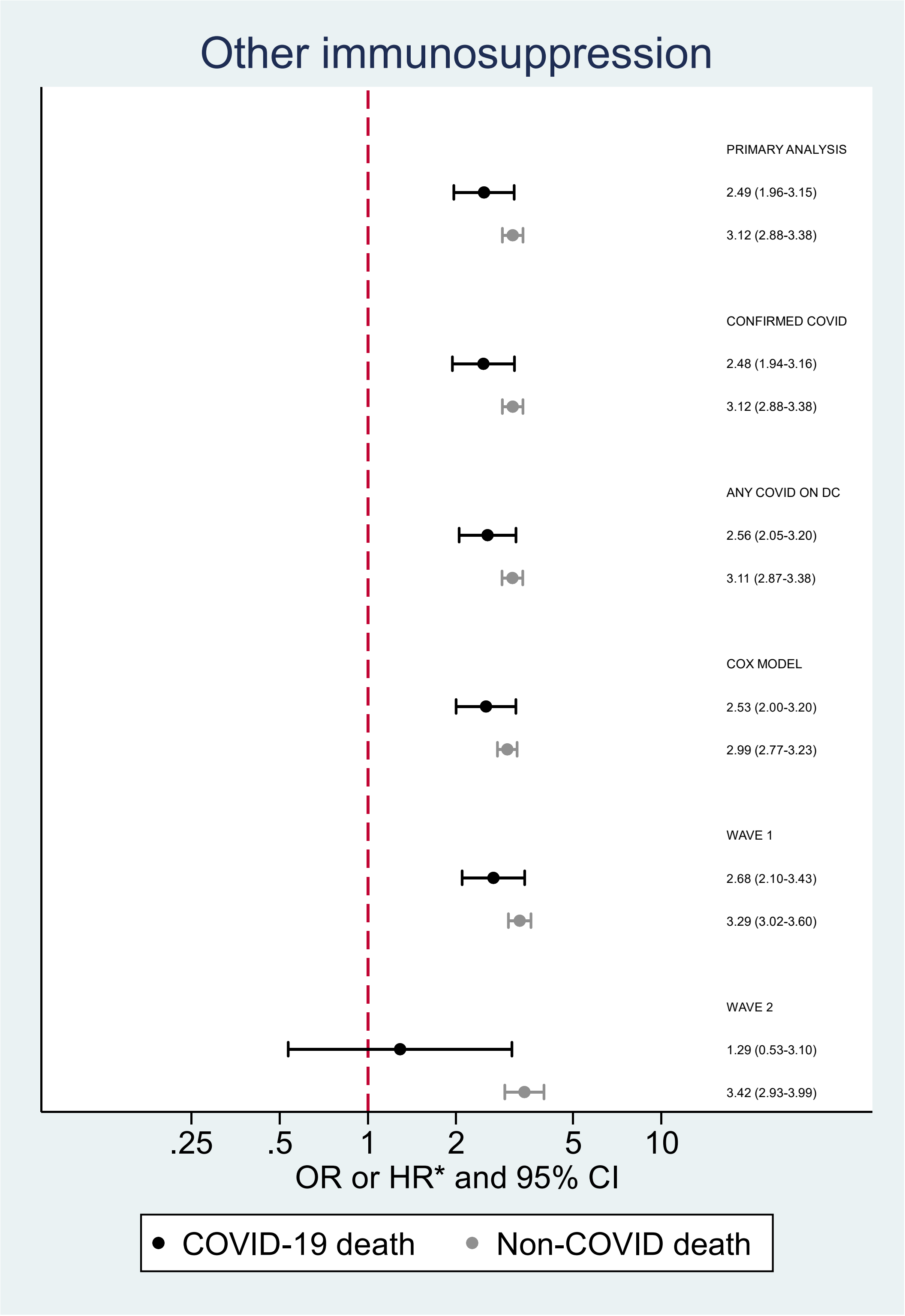

*CONFIRMED COVID = COVID-19 outcome defined using only the ICD-10 code U07.1 (COVID-19 – virus identified); ANY COVID ON DC = COVID-19 outcome defined as a COVID-19 code anywhere on the death certificate, rather than only as the underlying cause of death; COX MODEL = Hazard ratios from a Cox regression model (containing age / sex / covariate of interest and stratified by STP); WAVE 1 = 1st February to 31st August; WAVE 2 = 1st September to 9th November. *HR for the Cox model results, OR for all other results.*
